# Supplementary material for: Retrodicting the rise, spread, and fall of large-scale states in the Old World
Source: PLoS One. 2022 Jan 12;17(1):e0261816. doi: 10.1371/journal.pone.0261816 (PMC8754308; doi:10.1371/journal.pone.0261816)
Supplement: S1 File — (PDF) [file pone.0261816.s001.pdf]

Supplemental Information

Retrodicting the rise, spread, and fall of large-scale states in the Old World

James S. Bennett (jsb11@uw.edu)

The Historical Data

The model was developed and evaluated employing two data sources. The first dataset, a slightly-revised version of the dataset developed by [1] (here 'TCTG13'), describes the Afro-Eurasian continent as a gridded landscape of rectangular regions stretching about 2° of longitude and 1° of latitude and occupying about 20 thousand km^2 at the equator. Each region encodes its mean elevation and predominant 'biome type': desert, steppe, or agricultural. Each land region is connected to its immediate cardinal neighbors if they exist. Regions are marked as 'littoral' if any of their neighbors are an ocean or a sea. Desert regions that border agricultural 'oases' are also marked.

The dataset also encodes the regional locations and extent of all large-scale states ('centralized macro-states', as [1] define it) present at each century between 1500 BCE and 1500 CE in Afro-Eurasia (the 'Old World'). Each encoded state occupied at least 10 regions (about 200 thousand km^2, roughly the size of the contemporary Czech Republic or the historic Duchy of Burgundy). To compare the model results with the performance of [1] and [2], I measured polity sizes using the same region-count metric. The total area occupied in time by these large scale states, including nomadic confederations, are shown in the main paper, Fig 4A, as the black line; the total agricultural area occupied by agrarian states is shown in as the blue line in Fig 4A. The data depict roughly two major agrarian expansion periods (600 BCE to 100 CE and 700 CE to 1300 CE) between flanking periods of relatively little expansion. The waves of large-scale steppe nomadic states that pestered the agrarian states after 300 BCE are also clearly visible. These include the Xiongnu in 300 BCE, the Khazan in 700 CE, and the Golden Horde in 1300 CE.

The second dataset encodes spatially and temporally resolved population estimates of Kaplan, *et. al.*, [3] and [4] ('KK10'). This dataset contains yearly population estimates between 6050 BCE and 1850 CE. Based, in turn, on several well-known sources of historical population data (e.g., [5]), the population dataset is organized by modern, worldwide 'territories' (e.g., Paraguay, Minnesota, etc.). A separate territorial definition dataset, also supplied by Kaplan, provides latitude and longitude for each modern territory region resolved to .08 degrees. Combined, the KK10 datasets provided per-century estimates of total population in the Old World, which is shown in the main paper as the black line in Fig 4B. The population estimate shows a weakly quadratic rise from 70 million to 390 million in this period with some major episodes of plague and famine clearly present in the record, notably the Justinian pandemic and Chinese famines and plagues beginning around 500 CE and the Black Death in the mid-fourteenth century.

Together, these two datasets represent the most comprehensive and available to evaluate the model at this time.

The Model of State Dynamics

The sections below provide more detailed descriptions of the operation of agrarian state and nomadic tribes and confederations outlined in the main paper. Much of the discussion of agrarian

states is taken, unchanged, from [2]. The spatial and temporal behavior of the model under nominal operation can be seen in the accompanying animation. The movie depicts a single simulation run and employs a different colored marker randomly assigned to each polity to distinguish it for its duration; markers are reused.

As will be seen many (but not all) of the parameters, representing expected historical means in this period, were estimated using the primary datasets themselves. Prior sensitivity analysis discussed in ([2] Supplementary Information) indicates that most parameters admit a substantial range of values without disturbing the model behaviors. That analysis suggested that military efficiency s was a clear exception; this effort provides a more thorough investigation of the historically plausible events and scales that could modulate that parameter over time on a per-state basis. Nevertheless, finding independent ways of bounding or estimating all these (or similar) parameters represents avenues for future work. Although there are a relatively large number of direct parameters (21 for agrarian states, 12 for nomadic polities, controlling various sub-behaviors), they are used to estimate approximately 18 million data points per simulation.

Agrarian State Demographic Dynamics

To predict the agrarian state populations I employ the large-scale demographic model of [6]. The model assumes that an agrarian state, by its enhanced organization, is able to support greater crop yields, and hence carrying capacity advantage, over a single agricultural region. In particular, the model posits a simple, uniform threefold carrying-capacity advantage of multi-region agrarian ‘state’ polities over single region 'hinterland' polities between 1500 BCE and 1500 CE. The regional population P_r is grown logistically following Equation 1:

(1)
$$\dot{P}_r = \beta(\tau_r)\left(1 - \frac{P_r}{K_r}\right)P_r$$

where $\beta(\tau_r)$ is the net birth rate associated with the polity type τ (agrarian state or 'hinterland', defined above) in region r and K_r is its carrying capacity.

Regional carrying capacity estimates K_r follow Equation 2, which reflects mean population densities ρ for each polity type and an exogenous, temporally varying regional agricultural intensification schedule $I_r(t)$:

(2)
$$K_r = \rho(\tau_r)\, I_r(t)\, A_r$$

where $\rho(\tau_r)$ is the population density associated with the polity type τ in region r and A_r is its area. The values of $I_r(t)$ are shown in the main paper, Fig 1.

In cases where regional population exceeds carrying capacity ($P_r > K_r$), as happens when an state collapses to hinterland, the precipitous population collapse is modeled as

(3)
$$\dot{P}_r = -(P_r - \delta K_r)$$

where δ is the fraction of the carrying capacity to which the population falls. Bennett [6] found that integrating the predicted regional populations at each time step according to these equations and assumptions when the historical states encoded in the TCTG13 dataset changed regionally yielded a plausible prediction of the total population of the Old World as reported in KK10. Famines and plagues are not currently modeled.

Mean historical hinterland and state population density estimates and the state net birth rate are based on scaled population and agrarian yields from medieval England between 1150 CE and 1300 CE found in [7], a robust and independent analysis. The hinterland net birth rate is based on KK10 historical population data in regions without states. The birth rates reflect the typical net birth rates at low actual population densities for the two types of polities. The effective birth rate of Equation 1 depends on both these base rates and the residual carrying capacity available: As a growing population saturates regional carrying capacity, the effective net birth rate becomes a fraction of the base rates. While no doubt some 'positive checks' (delayed marriage, contraception, etc.) to population [8] increase in frequency as the residual carrying capacity diminishes, the number of children born likely continues apace, yet they (and the elderly) succumb due to insufficient nutrition, increasing the experienced misery for all.

The population densities estimates employed here do not reflect the total absolute production (say, as net primary production) required to support that population. Indeed, I estimate the true production of the land to be at least ten and more likely one hundred times higher in order to also supply seed for replanting as well as grain and fodder to fed various livestock, especially horses and cattle, each of which require roughly ten times the caloric intake as a human. However, the focus of this model is on the human population of agrarian states so this additional productivity is assumed and neglected.

The demographic model makes no assumptions about how agrarian polities rise and fall and thus can serve separately to predict the demographics that different imperiogenesis models generate.

Table 1. Agrarian state demographic parameters.

Parameter	Value
Agrarian state birth rate ($\boldsymbol{\beta_s}$)	1.35%/year
Hinterland birth rate ($\boldsymbol{\beta_h}$)	0.02%/year
Agrarian state population density ($\boldsymbol{\rho_s}$)	12 people/km^2
Hinterland population density ($\boldsymbol{\rho_h}$)	4 people/km^2
Collapse population loss fraction ($\boldsymbol{\delta}$)	80%

Modeled Agrarian State Dynamics: Conquest and Collapse

The population of agrarian states are composed of two sectors: a group of farmers working the fields to produce the food to support both themselves and a smaller group of warrior 'elites' who provide protection against banditry and annexation, as well as manage and invest the infrastructure (here people) required for production of food. Each region's population is split between the sectors and the region's total carrying capacity is divided between the sectors in a fixed fraction, ε. The constant ε, applied uniformly to all states, reflects a plausible guess of total elite fraction from historical estimates of medieval and ancient societal structures (see [9], for example). The particular value of ε serves largely to scale the size of projected armies (and their deaths).

The population of each sector grows separately and logistically per region, following Equation 1:

(4)
$$\dot{F_r} = \beta_F(\tau_r)\left(1 - \frac{F_r}{(1-\varepsilon)K_r}\right)F_r$$

(5)
$$\dot{E_r} = \beta_E(\tau_r)\left(1 - \frac{E_r}{\epsilon\, K_r}\right)E_r$$

where F_r and E_r are the current populations of a region's farmers and elites and $P_r = F_r + E_r$. Although a good case has been made that the elites would enjoy a higher net birth rate than the farmers, for simplicity I assume they are identical ($\beta_F(\tau_r) = \beta_E(\tau_r)$) and, following the demographic model, are equal to either β_e or β_h depending on the agrarian polity type. In the case of carrying capacity collapse, Equation 3 applies to each sector separately using the same collapse fraction δ.

The simple demographic model implements a within-polity migration scheme in which a fixed fraction of a polity's population moves between its regions each time step according to available residual carrying capacity. For agrarian states, the warriors and peasants have separate migration fractions $\mu_\varepsilon$and μ_P. Fraction values for population migration and death from carrying-capacity collapse were estimated from overall fit of the model to TCTG13 historic polity and KK10 population data.

The initial (possibly discounted) KK10 historical population in 1500 BCE is divided regionally according to initial agrarian polity type (hinterland or starting states), and each region's population is then divided into farmers and elites according to ε.

Armies and Annexation

As the number of elites increases via Equation 5, their ability to militarily project their power increases and they become able to annex additional neighboring agricultural regions. However, power projection is subject to its own (spatial) logistical constraints as it takes more people in the elite entourage to support an army prosecuting a battle in a distant neighboring territory. In particular, I assume that all the polity's elites are effectively concentrated into a central depot and then spread out to various political borders. The force, *H*, that can be projected to a contested region is given by:

(6)
$$H_{polity}(d) = \left(\frac{\sum_{polity} E_r}{\left|Borders_{polity}\right|}\right)L(d)$$

(7)
$$L(d) = \frac{1}{\left(1 + \frac{1}{s}\right)^{d}}$$

where $H_{polity}(d)$ is the size of the polity's army at the region *d* kilometers away from the polity's depot, $\sum_{polity} E_r$ is the elite population of the entire polity and $|Borders_{polity}|$ is the number of polity regions that have *political* borders requiring defense or a battle to annex. I assume *physical* border regions such as oceans and impenetrable mountains require a negligible defensive army. The location of the polity's depot is chosen as its geographic center at the formation of the polity.

The function $L(d)$ encodes how projected military power attenuates with distance. As supply lines lengthen from the depot to the contested border, some part of the army must support the soldiers doing the fighting. As distance increases, those support troops themselves need support, etc., leading to the recursive form of $L(d)$ in Equation 7 above ([10]; see [2], Supplemental Information). The key logistic scaling parameter, *s*, is the number of fighting soldiers supported per supply soldier per 100 km; larger s implies a larger projected force at a given distance. Distances can depend on sea- and desert-based discounted distances as computed below.

At each time step every agrarian state decides whether to launch offensive attacks on some of its neighbors. All immediate agricultural, political neighboring regions bordering the state are inspected, preferring regions with a minimum expected productivity I_{min}. Since the state population density value is fixed (ρ_s), the expected productivity is reflected by the regional intensification factor $I_r(t)$ in Equation 2. If the intensification factor of the targeted region is below I_{min} the region is deemed 'not worth it' and the order is not written. The productivity values for high elevations (above 3 km) are also very small, ensuring that states avoid annexing these regions. At most 45% of the preferred border regions (the 'attack budget') are then stochastically chosen and 'orders' to attack these regions are written. The attack budget effectively limits the total number of attack orders written per polity each time step. At each time step, all the orders from all the polities, including the nomads (see below), are collected, randomly shuffled, and then executed. Shuffling all orders ensures there is no overall spatial bias to polity growth.

When each annexation order is executed it is first checked that the offensive polity still occupies the attacking region (they could have been attacked themselves and annexed) and that the attacked region is still occupied by the same 'other' polity when the order was written. If not, the order is rescinded. Otherwise the projected power of the polity armies at the attacked region is assessed using Equation 6 and if the ratio of the offensive to defensive power is greater than a threshold, Δ, the attacked region is annexed by the victorious polity.

(8)
$$\frac{H_{attacker}(d_{attack})}{H_{defender}(d_{defend})} \geq \Delta$$

where d_{attack} is the distance from the attacker's depot to the chosen region and d_{defend} is the distance from the defender's depot to the same region. Larger values of Δ require larger elite populations (for a given s) to achieve the same territorial expansion, which interacts with elite immiseration described below.

When a battle is successfully waged, some fraction of both the victorious and the vanquished *projected* elite armies *H* at the battle site are killed. Each elite army is formed by contributions (here assumed to be 100%) from all the polity's elites in all regions, which are then

redistributed to the contested borders according to Equation 6. Therefore any battle deaths must be made proportionally to elite population distribution over *all* the polity's regions, not just within the contested region. In this way battles, especially unsuccessful ones, can slowly weaken the entire polity and eventually its ability to support its borders, even causing runaway loss of all territories.

The two death fractions, $\delta_{victorious}$ and $\delta_{vanquished}$, are set to different values (see Table 2), arrived at empirically by varying these parameters leaving all else fixed and observing that the overall predicted demographics of the world remained plausible. In the current model, there are no deaths of farmers on either side during battles, nor is there loss of agricultural productivity. While it would be straightforward to add more death fractions for the farmers similar to the elites (except they would apply locally), I elected not to complicate the model and assume, instead, that the vanquished farmers continue to work under the victorious elites.

Immiseration, Collapse, and Civil War

Territorial expansion continues until the spatial logistic limit set by s (and Δ) is reached and the growth of elites is constrained both spatially and by carrying capacity limits in the occupied regions. According to demographic-structural theory, as these limits to growth are approached, the elites become immiserated, cease their cooperative behavior, and engage in intra-elite competition. The farmer population, of course, has been growing as well and their opportunities have also been shrinking. The theory predicts many consequences of this farmer immiseration such as real wage loss, reduced health as diets become poorer, rural flight to urban locations, and food riots. I model none of this and assume that in spite of the elites own increasing immiseration they retain enough cohesion to suppress any insurrections by the farmers. Thus, farmers come to saturate their fraction of the carrying capacity, but their fate remains controlled by the elites.

Whenever a region's consumed elite opportunity $\left(1 - \frac{E_r}{\epsilon K_r}\right)$ exceeds a threshold Θ the state's elites rebel and engage in civil war. Goldstone [11] suggests that some (large) fraction of a polity's elites must become miserable before a rebellion occurs, but I model the trigger on a single region's elites. Empirically this appears to be a plausible assumption, because most regions are annexed early around the same time and grow at the same rate thus most regions are not far behind one another in saturating their opportunities.

In the model reported here, once a rebellion is raised, the polity collapses into two new factional states. To form these factions, two of the collapsing polity's border regions are chosen at random and assigned to new faction polities. Then, in a round-robin fashion, each faction chooses a region on its expanding border, regardless of its power, that was part of the original polity but has not yet been assigned to another faction. A faction occupying a single region is converted to hinterland. Once the cohesive factions are formed they operate as usual and prosecute annexation wars, often against their opposing faction, since by construction they share an extensive political border. These civil wars are fought identically to normal annexation wars between different polities.

Reviewing the historical data in TCTG13, portions of states sometimes collapse below the minimum polity scale (10 regions) and appear to revert to hinterland. However, I model this as a chance for a new faction to itself immediately collapse to another set of factions. I found all episodes in TCTG13 where an state lost at least 10% of its prior century's territory and then computed the fraction of the actual loss (often much higher) that went to apparent 'hinterland' (as opposed to other large states). The odds that more than 50% of lost territory collapsed to smaller states are about 25% (76 out of 294 loss events). Based on this value, I set the odds (f_h) for a

formed faction to fall immediately to another set of factions to 25%. Falling to new factions causes population loss for both factions (following Equation 3) and eases the field for surrounding states, including other factions, to rapidly annex this territory.

Factions are formed because of a succession by (at least) one immiserated region. The split into factions does nothing to reduce the misery of that region and any faction that inherits it would itself immediately collapse, recursively, until that region was reduced to hinterland. To avoid this either elite opportunity or the elite's tolerance for misery must temporarily increase; I model a mixture of both. I chose a 'grace period' (g) of 25 years, one generation, during which the factions might stabilize into longer-lived polities (or fall again). I achieved this both by eliminating a fraction of elites from each faction (assumed to perish during the spasm of collapse) and by raising the misery threshold for all factions for that period of time, in some combination controlled by a mixing parameter, *c*. The increased misery threshold Θ' reflects a temporarily increased cooperation by *both* factions to their new respective causes. The new misery threshold, Θ', and the drop in elite population given *c* and *g*, can be estimated using Equation 1 (see [2], Supplemental Information). I chose *c* to be 80% death, 20% increased tolerance, implying a Θ' of 85.8% (a 1% increase in tolerance) and a drop in the elite population to 86% of εK. After adjusting the elite population, a timer is set on each factional polity for the grace period; once it expires, the polity's misery threshold is reset to Θ, reflecting a loss of heightened support to the faction and perhaps a desire to return to normal life. Any regions that exceed Θ at that point (or subsequently) will trigger a collapse of the polity.

Table 2. Agrarian state parameters.

Parameter	Value
Elite carrying capacity fraction ($\boldsymbol{\varepsilon}$)	5%
'Worthwhile' agricultural productivity ($\boldsymbol{I_{min}}$)	0.6
Elite migration rate ($\boldsymbol{\mu_{\varepsilon}}$)	0.01%/year
Peasant migration rate ($\boldsymbol{\mu_P}$)	0.10%/year
Soldiers per supply soldier per 100 km (*s*)	1.1 initially, 2.1 at most
State attack budget	45% of annexable regions
Victory power ratio ($\boldsymbol{\Delta}$)	1.2
Death fraction for victorious elites ($\boldsymbol{\delta_{victorious}}$)	10%
Death fraction for vanquished elites ($\boldsymbol{\delta_{vanquished}}$)	80%
Military efficiency adaptation rate ($\propto$)	0.1/year
Collapse threshold ($\boldsymbol{\Theta}$)	85% of regional elite opportunity
Grace period after collapse (*g*)	25 years
Death/tolerance mixing parameter (*c*)	80% death
Number of factions per collapse	2
Chance of a faction collapsing again ($\boldsymbol{f_h}$)	25%

Nomadic Tribal and Confederation Dynamics

The model of the steppe nomads is extremely simplified. At the beginning of the simulation, the (extended) steppe region (see Fig 1) is stochastically populated by ~50 nomadic tribes. For each tribe, its neighboring and the set of agricultural regions within the tribal and confederation striking ranges, if any, are computed and cached for the duration of the simulation, for use during extortion

calculations. These distances for steppe nomads are always Euclidean; discounted sea and desert connections are ignored.

Initially nomadic tribes have no impact on agrarian states (or each other). However at some point, horse cavalry is invented by tribes near a specified longitude (nominally 45°E for the Pontic-Caspian steppe region) in a specified year (nominally 1000 BCE). The model maintains two frontier limits, one west of the starting longitude, the other east. Each time step, these frontier limits are advanced to the west and east, respectively, by an amount such that the distance from the Pontic-Caspian region to China would have been filled in a specified amount of time. As these frontier limits advance, any nomadic tribe within these frontiers adopts horse cavalry, until all nomadic tribes have adopted it. The starting longitude, time of invention, and time to propagate are specifications to the model and permit counter-factual experimentation with alternative nomadic spread scenarios. The nominal values reflect data reported in [12]; see their Figure 1.

Once a tribe adopts horse cavalry they become able to extort any agrarian states that expose some fraction of their territory (10%) within the tribal striking distance. Tribes, for example, in the Siberian north or in the middle of the steppe range, have no agricultural regions within reach and cannot extort any state. However, tribes that are near the borders of agricultural lands may find agrarian states to menace. If so they are placed on a list of states the tribes extort and the agrarian state which tribes are extorting it. Once a tribe is able to extort it is always able to extort. A tribe (or confederation) can extort several agrarian states; an agrarian state can be extorted by several nomadic polities. When an extorted agrarian state collapses it is no longer extorted (and is removed from the extorting nomadic polities). Any newly-formed agrarian states will be evaluated for extortion by the nomads at the next time step.

Extortion has a number of un-modelled effects, such as increasing trade between agrarian and nomadic polities. Here, however, the only modelled effect is to present a significant military threat to the extorted agrarian states. Their response, and the subsequent response of the nomadic polities is outlined in the section below.

As the extorted agrarian states increase in size and population the fraction of extorted territory of these states decreases. To maintain sufficient territorial targets for effective extortions of these more wealthy (i.e., more populous) states nearby nomadic tribes confederate to increase their logistical ability to strike deeper into these agrarian states. To form a confederation, an extorting tribe waits until the total extorted population of all states within its tribal strike distance exceeds a certain threshold. Once that threshold is crossed, the tribe determines if there are minimum number of adjacent tribes that are not already part of a confederation, regardless of whether they are extorting states themselves. These 'inner' tribes then join together to form the initial nucleus of a new confederation. The individual tribes no longer extort the agrarian states; the confederation extorts instead employing the expanded strike distance, allowing more territories (and possibly additional agrarian states) come under the sway of the confederation. Like nomadic tribes, confederations continue to expose the agrarian states to increased military threats and the state continues to adjust accordingly.

As the extorted agrarian population (and perhaps size) increases the steppe nomadic confederation expands its size as well by recruiting adjacent 'outer' tribes not already part of another confederation. I limited the size of a confederation to be roughly one-half of the tribes since that appears to account for the maximum size of confederations in the steppe area late in the TCTG13 historical record. Note that using the Mongolian *yam* postal system, a mounted messenger could cover 200-300 km/day which implies a cross steppe communication time of roughly 20 days, or 10 days for half the steppe.

The steppe/agrarian geography limits the number of locations where modeled nomadic confederations can form. Tribes, which are territorially large themselves, must have access to agrarian states within a tribal strike zone and must also be connected to other nearby tribes not already part of a confederation. The presence of agrarian states in the model is constrained by the underlying productivity of the land, which changes exogenously during the simulation. Thus, in spite of the long (eventual) tribal strike zones shown in Fig 2 of the main paper, these constraints restrict confederation formation prior to 300 CE to the frontier in the north and northwest of China, in the Transoxiana region near the Caspian Sea, and the Pontic-Caspian region. After 300 CE the eastern European frontier to the west of the Urals comes to host additional confederations as the adjacent agricultural land become more productive and conducive to agrarian state formation (Fig 2B).

If the extorted agrarian population falls below the confederation threshold (for example, by state collapse and civil war), the confederation also collapses. Historically, confederations suffered recurrent lateral succession crises among the related leaders of the confederation. I model these conflicts as a simple odds of collapse once every few generations. Thus each generation (g, 25 years as with agrarian states) since the founding of the confederation the model tests for a succession crisis. When a collapse occurs, the confederation is dissolved into its constituent nomadic tribes, which revert to extortion at the tribal strike distance.

Historically the steppe nomadic population varied between 1 and 4 million in this period, a small fraction of the total Old World population. The model currently does not track the nomadic population. Unlike agrarian states, whose populations drive their influence and fluctuate with productivity, I assume the nomads (and their livestock) live at or near the carrying capacity of the steppe and that any demographic losses because of inter-tribal warfare or extreme weather fluctuations (e.g., *zud*) are transient and can be neglected and that there is always sufficient force to successfully extort any agrarian states that are within striking distance. Individual attacks of nomads against agrarian states, unlike agrarian inter-state annexation battles, are not modelled and neither agrarian nor nomadic polity types attempt to conquer the other.

Increasing military efficiency under extortionary threat

In the model, agrarian states increase their general military efficiency (s) in response to increased nomadic 'effective s' (s_n). At each time step, an extorting nomadic polity (tribe or confederation) determines the maximum s of any of its extorted agrarian states (the most powerful state) and if that value exceeds 95% of the nomadic polity's s_n, a small increment is added to s_n up to a simulation maximum. Since the nomads were able to maintain military dominance in this period I assume they are always able to present such a slightly elevated s_n, maintaining their superiority. Formation of nomadic confederations, with their deeper logistical reserves, is no doubt one of the ways this dominance was maintained.

On a subsequent time step, each agrarian state determines the maximum s of any neighboring agrarian state and the s_n of any extorting nomadic polities. If the maximum s (s_{max}) is greater than the state's current s, the agrarian state increases its s, also bounded by the simulation limit, according to the following equation:

(9) $$\Delta s = \alpha(s_{max} - s)$$

Thus the greater the difference between military efficiencies, the faster the increase of an agrarian state's s. Adjusting the parameter α permits exploration of impact of different rates of

increase on the timing and distribution of states. The nominal value of α permits an initial state to develop the maximum military efficiency in roughly two generations with over half the improvement occurring within a decade. Following demographic-structural crises, factions maintain their parent states' value for s.

Historically, agrarian states typically increased their military efficiencies by adopting horse cavalry themselves, often fueled by horses traded off the steppe. This adoption can be seen by combining the horse cavalry spread data from [12] with the TCTG13 historical state data, providing an estimate of the number of years before or after the first large-scale state appears at a reported horse cavalry location in time. The clear spike around 0 years shown in Fig S1 suggests that horse cavalry spread in Eurasia largely as agrarian states themselves spread.

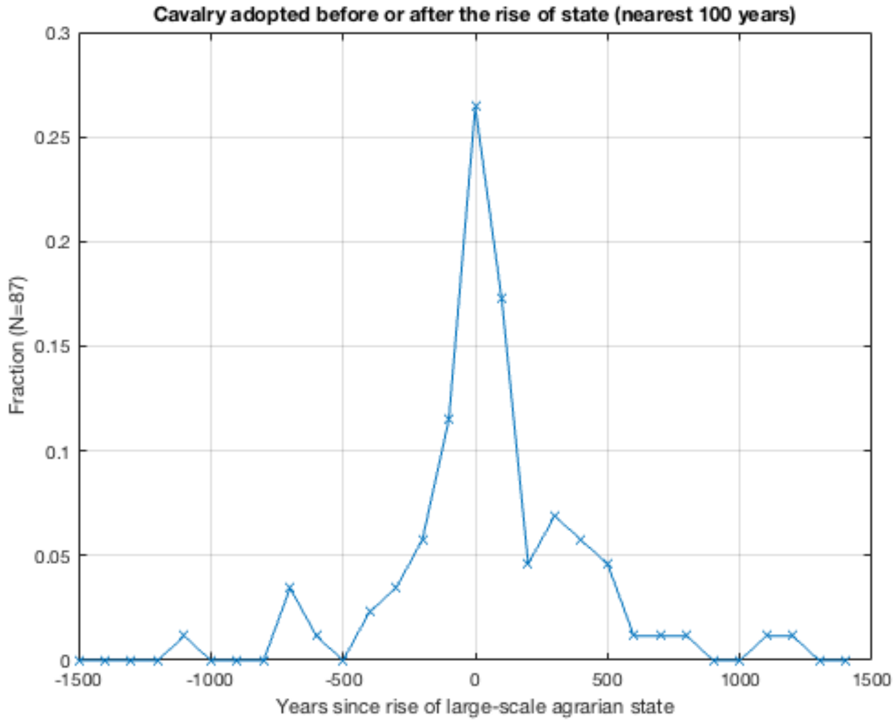

Fig S1: The fraction of first reported horse cavalry adoption points in [12] as a function of the time of the first state occupying the reported location, using TCTG13 state data. Negative years imply early adoption of horse cavalry before a large-scale state occupies that region.

Table 3. Nomadic tribe and confederation extortion parameters.

Parameter	Value
Nomadic tribe strike zone distance	200 km
Nomadic confederation strike zone distance	550 km
Nomadic confederation population trigger	6.2M people extorted
Minimum agrarian state area in strike zone	10%
Horse cavalry diffusion time across steppe	500 years
Maximum s_n upon adopting horse cavalry	1.4
Incremental s_n when state s increases	0.05
Maximum confederation s_n	2.1
Minimum number of confederation 'inner' tribes	5
Population increase to add an 'outer' tribe	19M people
Maximum number of tribes per confederation	25
Odds of lateral succession confederation collapse	Once every 6 generations

Exogenous contingencies

The agents above respond to a set of exogenous, contingent regional events and technologies, which can be modified to explore counterfactual simulation histories. For convenience, the nominal set of contingencies are summarized below.

- **Where and when initial states arise and their scale:** Small (2-3 region) states are initially seeded in the Nile valley and the Fertile Crescent areas in 1500 BCE. In 1200 BCE, another small state is seeded in the middle Yellow River valley in east Asia.
- **Where and when various agricultural regions change their productivities:** Agricultural regions change their productivity in time according to Fig 1 in the main paper.
- **Where and when horse cavalry was invented and the speed of its diffusion across the steppe:** Invention occurs by simulated nomadic tribes near 45°E in 1000 BCE and diffuses longitudinally to all remaining tribes in 500 years.
- **Where and when long-distance transportation is available at different efficiencies:** Sea-borne travel is limited to the Mediterranean; it is discounted by 30% (see below) and the permitted distances change from 223 km in 1500 BCE to 447 km in 1000 BCE and finally 670 km (roughly the distance from Carthage to Rome) in 500 BCE. Desert-based travel is limited to the Near East; it is discounted by 30% and the permitted distance changes from 200 km in 1500 BCE to 400 km in 900 BCE. Distance limits were estimated empirically. For locations, see Fig S2; for impact of discount factors on nominal simulations see Fig 7D and 8D in the main paper.

Computing discounted distances for agrarian states

Following [1] and [2], when computing possible annexations for agrarian states, if a littoral border region of a polity selects an adjacent sea region, a distant, connected littoral region within a given distance, if any, is randomly selected. This scheme was extended here to apply to desert regions: When an agricultural 'oasis' region selects an adjacent desert region, a distant, connected 'oasis' region within a given distance, if any, is randomly selected. Assuming the selected region is agricultural and belongs to a different polity, an order is written against it and pursued in the usual fashion. See main text for a discussion of the effect of this feature on model performance.

However, unlike [1] or [2], the power projection calculations here employ a potentially discounted distance from the depot to the attacked region, computed as described below. As noted above, for these simulations, sea-based battles are limited to the Mediterranean and desert-based battles are limited to the Near East. While pirates no doubt plundered all the seas, for example, near the Malacca Straits or along the eastern African and Asian seaboards, my interest is in agrarian states that employed sea power to annex and secure agrarian territory under their sway. These thalassocratic polities appear to be concentrated in the Mediterranean during this period. A similar argument can be made for desert-encompassing agrarian polities in the Near East.

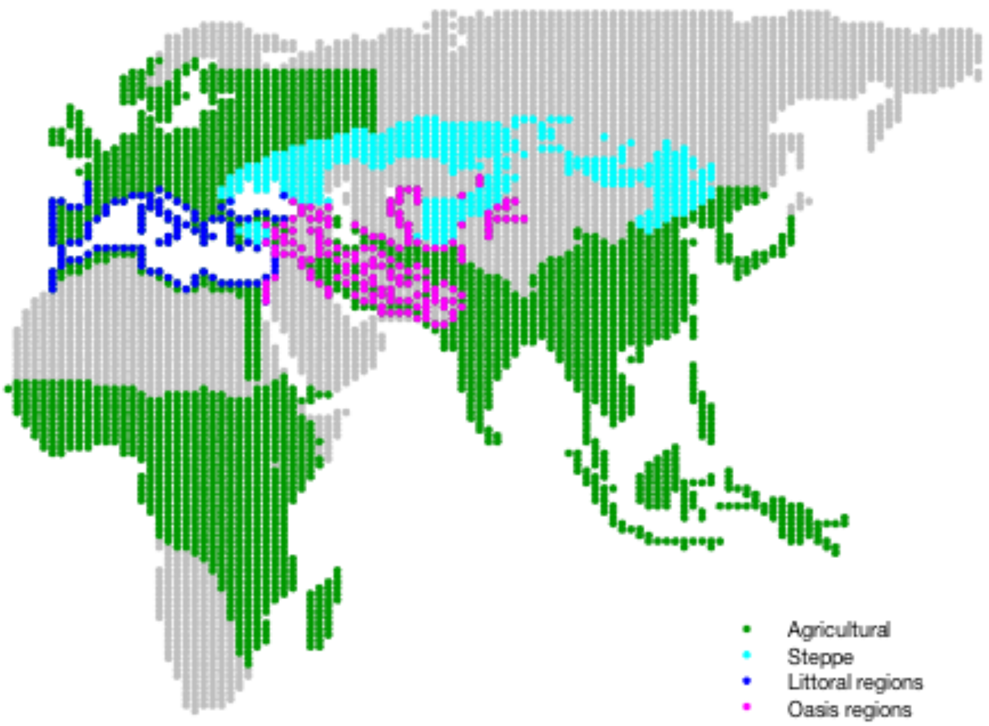

Fig S2: Location of littoral and desert 'oases' regions used in the nominal simulations. Map and biome data republished from [1] under a CC BY license, with permission from Proceedings of the National Academy of Sciences, original copyright 2013.

To compute the minimum effective distances from a state's depot, the algorithm first computes the direct Euclidean distance from its depot to all regions (under the assumption that all access will be via land connections with undiscounted distances). Then given all sea and desert 'embarkation' regions, it adds the current best distance from the depot to the embarkation point to the discounted distance between each embarkation point and each of its connected 'arrival' regions, according to the type of travel. If this discounted distance from the depot to the arrival region is shorter than the current best distance estimate to the arrival point, it then computes the land-based Euclidean distance from the arrival region to all other regions and adds this to the discounted stance to the arrival region. It updates the distance from the depot to any point if it is shorter via this combined path than via the prevailing best estimate (this will include the arrival region). Since the algorithm explores all possible sea and desert connections it ensures that multiple sea and desert combinations are eventually evaluated and the overall minimum distance is found. In the current implementation, these minimum distances are computed at the formation of the state when its depot location is fixed and are recomputed whenever the discount efficiencies change.

These per-state minimum distances are used by the state when computing projected military power (Equation 6). If either discount factor is set to 1 (no discount) then there is no distance advantage along that kind of connection. However, distant regions within the distance limit are still accessible if the embarkation region is annexed, even if the logistical distance is not reduced. This corresponds to the operation of sea-based connections in [2].

Results from Counter-factual Investigations

The following sections present details from the counter-factual investigations discussed in the main paper. Many of the investigations show an impact on the number of states, the area occupied by states over time, and different locations of expansions of states as indicated by a lower spatial R^2 metric.

Late adoption of camels for Near East desert military operation

Historically camels were employed for military purposes in the Near East around 900 BCE, the date used in the nominal simulations. However, if that technology had been adopted 300 years later, in 600 BCE there are various regional impacts on events. First, the delay ensures that the early Near East states will be smaller: Their reach is neither as far nor as efficient in spite of the small increase in s after nomadic tribes threaten them after 1000 BCE. Second, when the first nomadic confederation does arise in 500 BCE , still in the Pontic Caspian steppe region, the subsequent agrarian states, although powerful, are delayed in their invasion into Europe and India, reducing their imperial densities there.

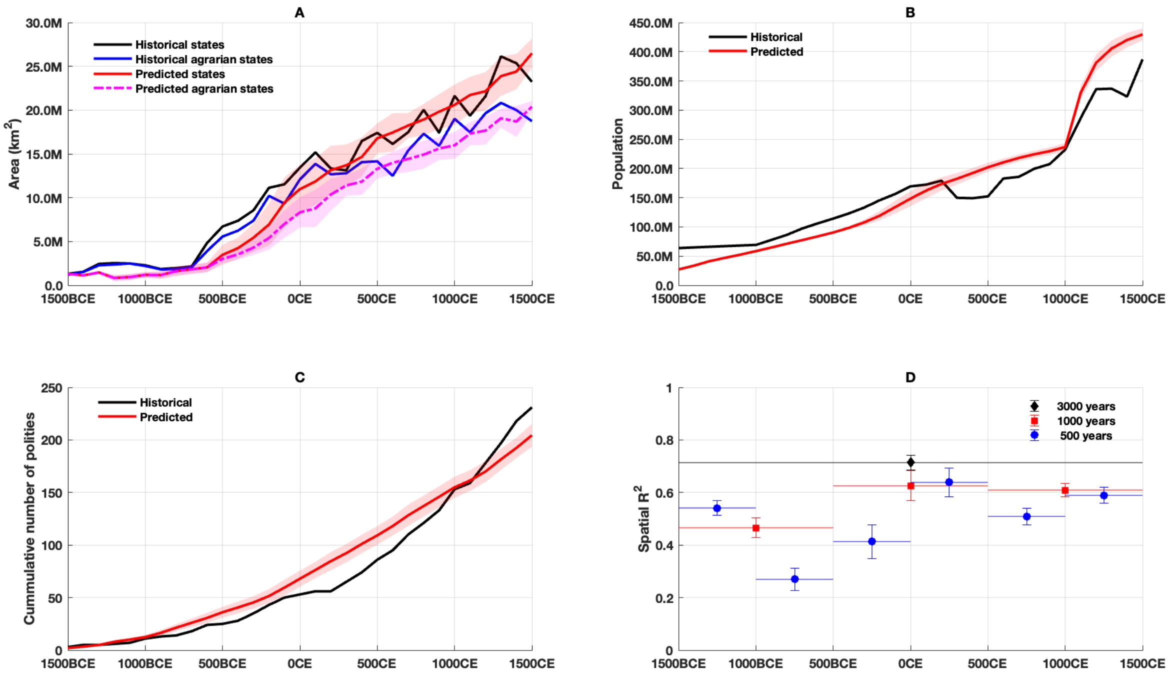

Fig S3: Predictions for the Old World with late adoption of camels in 600 BCE and confederation trigger of 6M based on 32 trials. **A**: Predicted mean and standard deviation of total area under all large-scale polities (red) and agrarian states (magenta) compared with historical values per century from TCTG13 (black and blue, respectively). **B**: Predicted mean and standard deviation of total population (red) compared with historical values every 20 years from KK10 (black). **C**: Predicted mean and standard deviation of cumulative (red) number of large-scale polities surviving a century compared with historical values (black) from the TCTG13 dataset. **D**: Mean and standard deviation of spatial R^2 metrics for 500 (blue), 1000 (red) and 3000 (black) year intervals. Compare predictions with Fig 4 in the main paper.

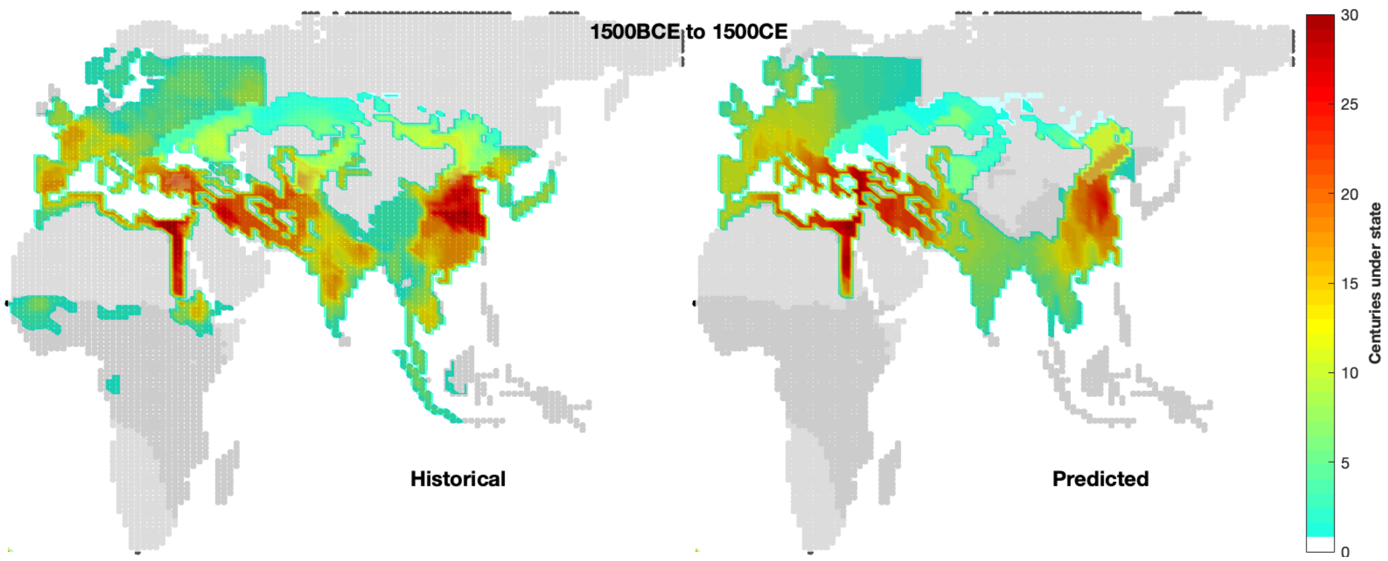

Fig S4: Mean large-scale polity density over 32 trials for the Old World for 3000 years beginning in 1500 BCE. Left panels reflect historical data from TCTG13 for large-scale states (≥ 10 regions); right panels reflect model predictions assuming late adoption of camels in 600 BCE and confederation trigger of 6M. Darker grey indicates available agricultural regions in the period; light blue indicates steppe. Compare predictions with Fig 3D in the main paper. Map, biome and historical data republished from [1] under a CC BY license, with permission from Proceedings of the National Academy of Sciences, original copyright 2013.

To ensure that a nomadic confederation arises roughly in 600 BCE (to correspond, again, to the historic Achaemenid/Scythia pair), the trigger threshold must drop to 4 million people (from 6 million nominally). This lower trigger value implies, however, that when nomadic horse cavalry arrives on the Chinese border in 500 BCE, the combined Chinese population in the (small) states around the middle Yellow River valley have long exceeded the trigger value (I estimate Chinese states exceed 4 million people in the tribal strike zones around 800 BCE) and an eastern nomadic confederation arises immediately, prematurely by 300 years, bypassing the phase of lower nomadic tribal threat (as can be seen in the bulge in Fig S5A around 300 BCE compared with the nominal run in Fig 4A in the main paper). Nevertheless, the overall long-term statistics, shown below, remain excellent.

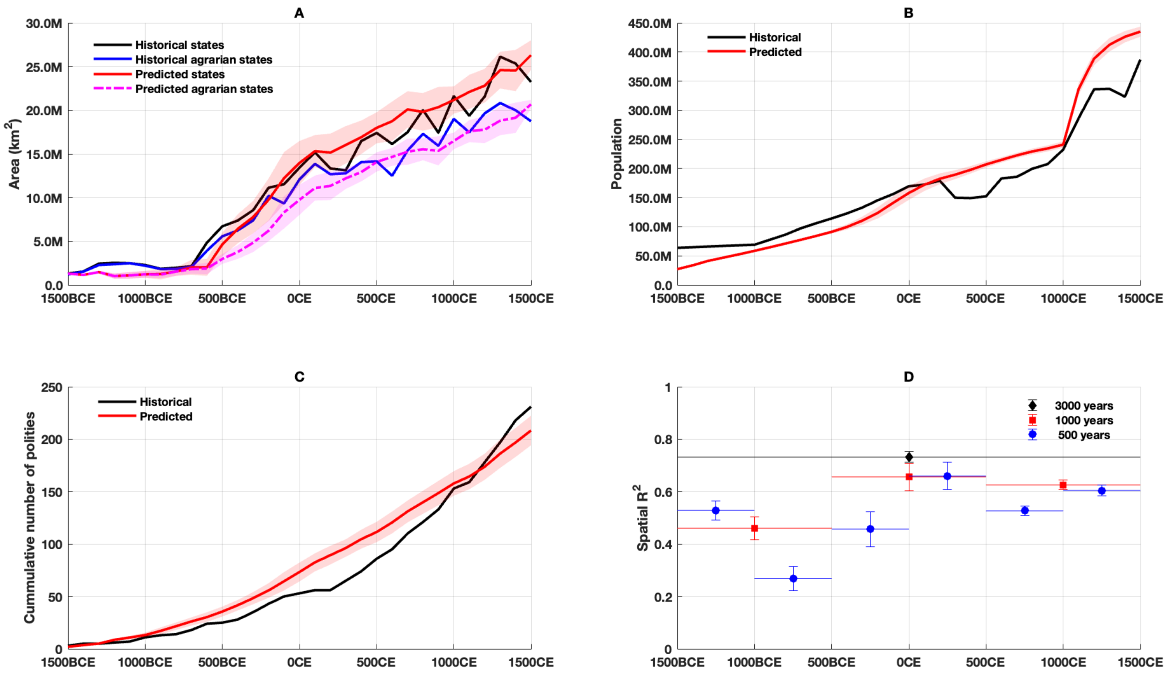

Fig S5: Predictions for the Old World with late adoption of camels in 600 BCE and confederation trigger of 4M based on 32 trials. **A**: Predicted mean and standard deviation of total area under all large-scale polities (red) and agrarian states (magenta) compared with historical values per century from TCTG13 (black and blue, respectively). **B**: Predicted mean and standard deviation of total population (red) compared with historical values every 20 years from KK10 (black). **C**: Predicted mean and standard deviation of cumulative (red) number of large-scale polities surviving a century

compared with historical values (black) from the TCTG13 dataset. **D**: Mean and standard deviation of spatial R^2 metrics for 500 (blue), 1000 (red) and 3000 (black) year intervals. Compare predictions with Fig 4 in the main paper.

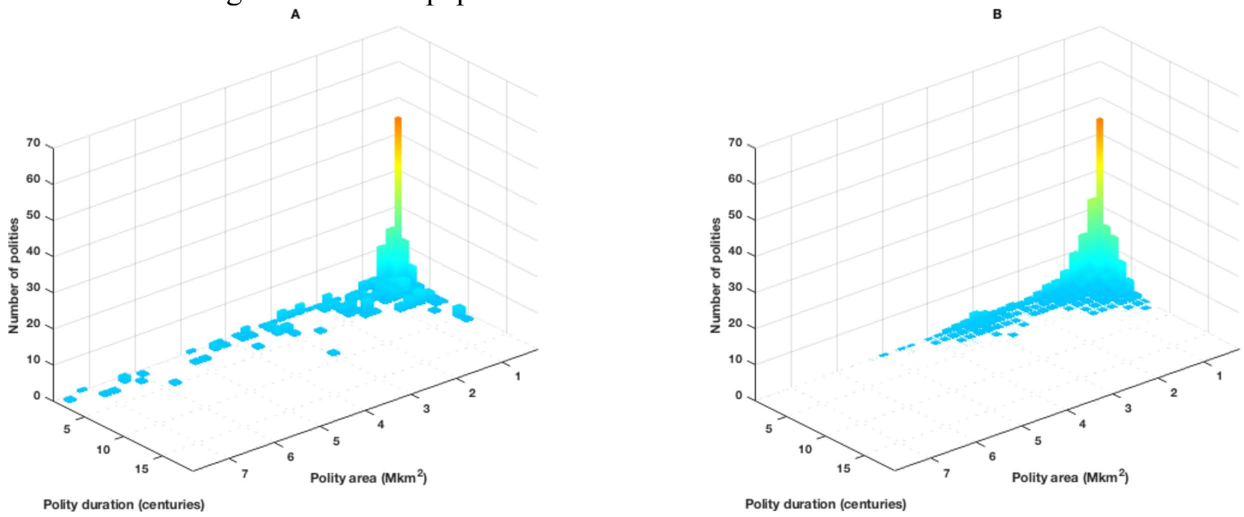

Fig S6: Historical (**A**) and mean predicted (**B**) distributions of peak (agrarian and nomadic) duration (in centuries) and peak size (in millions (M) of km^2) between 1500 BCE and 1500 CE assuming the late adoption of camels in 600 BCE and confederation trigger of 4M. Compare predictions with Fig 5 in the main paper.

Alternative Starting Agrarian States: Ganges and Mekong River valleys

If states arise further from the site of the eventual nomadic threat, the growth of states is of course delayed. In the experiment below, states are assumed to form first in the Ganges and Mekong River valleys. Further, all agricultural regions in east Asia must be assumed to be productive from 1500 BCE. During the first 1000-year interval the (weak) states from the Ganges eventually diffuse west (they are checked in the east by the states in the Mekong) and then, because of efficient desert transport, expand rapidly toward the nomads. Confederations form in the Transoxiana region around 0 CE. In spite of their increased s these central Asian states are delayed across the deserts of the near East and, in most simulations, never enter Europe by 1500 CE. However, and more surprising, the states stretching back to the Ganges also become increasingly powerful (their s increases in a 'boomerang' wave to the south east) but no consolidation occurs as those long-existing states in southeast Asia effectively check one another. However, beginning in 500 CE, the wave of increased s reaches the small-scale states originally from the Mekong, hitherto weak and slowly expanding north, and they now expand in size and rapidly invade China from the south, eventually triggering eastern confederations after 1000 CE.

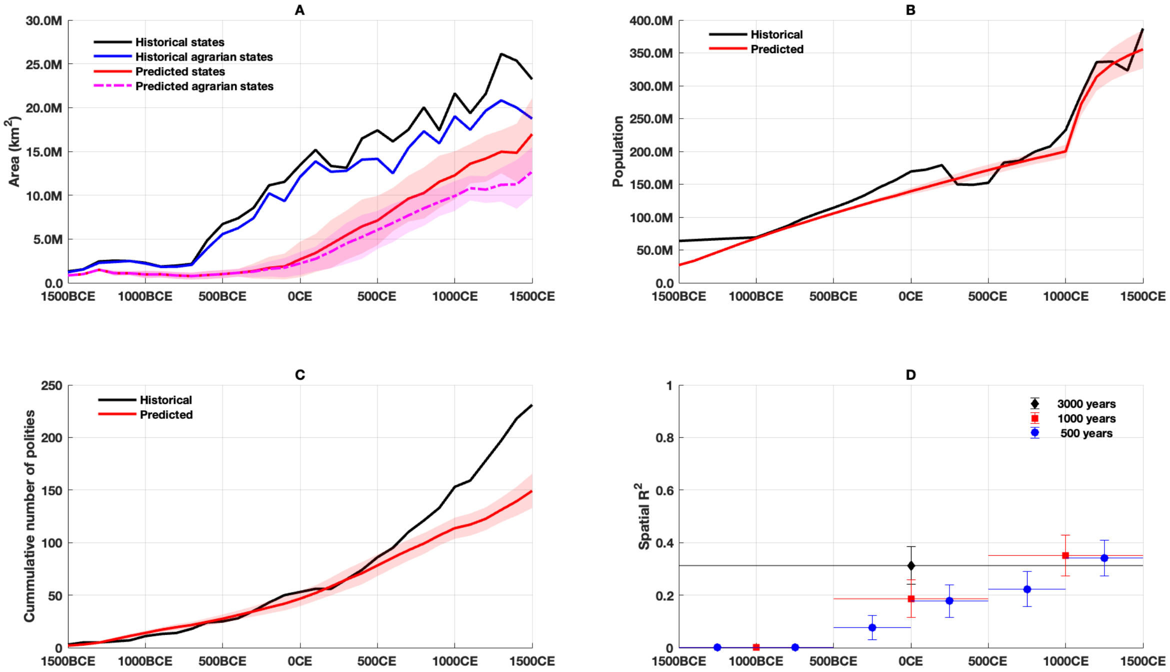

Fig S7: Predictions for the Old World with initial polities in the Ganges and Mekong river valleys based on 32 trials. **A**: Predicted mean and standard deviation of total area under all large-scale polities (red) and agrarian states (magenta) compared with historical values per century from TCTG13 (black and blue, respectively). **B**: Predicted mean and standard deviation of total population (red) compared with historical values every 20 years from KK10 (black). **C**: Predicted mean and standard deviation of cumulative (red) number of large-scale polities surviving a century compared with historical values (black) from the TCTG13 dataset. **D**: Mean and standard deviation of spatial R^2 metrics for 500 (blue), 1000 (red) and 3000 (black) year intervals. Compare predictions with Fig 4 in the main paper.

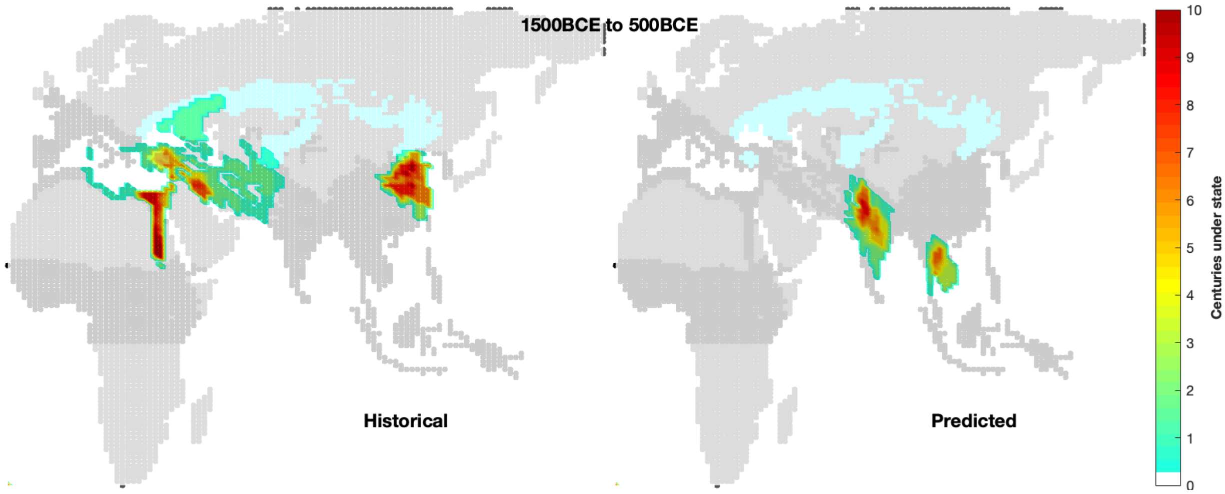

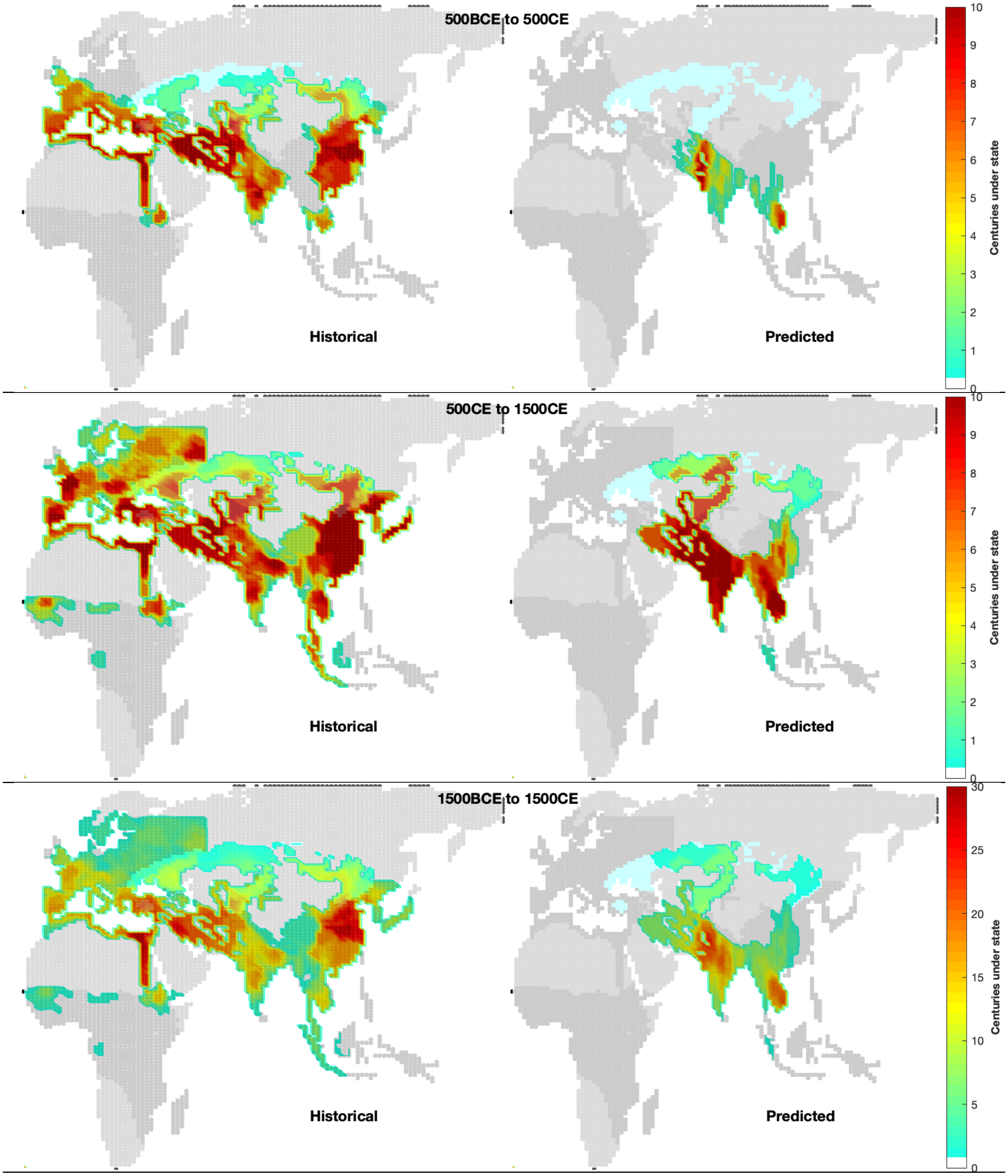

Fig S8: Mean large-scale polity density over 32 trials for the Old World at the end of each 1000-year interval (**A**, **B**, and **C**) and over 3000 years (**D**) beginning in 1500 BCE. Left panels reflect historical data from TCTG13 for large-scale states (≥ 10 regions); right panels reflect model predictions assuming initial polities in the Ganges and Mekong river valleys. Darker grey indicates available agricultural regions in each period; light blue indicates steppe. Compare predictions with Fig 3 in the main paper. Map, biome and historical data republished from [1]

under a CC BY license, with permission from Proceedings of the National Academy of Sciences, original copyright 2013.

Of course there are many more smaller states that are not invaded and so last slightly longer in these simulations, consistent with the dynamics outlined above (Fig S7C).

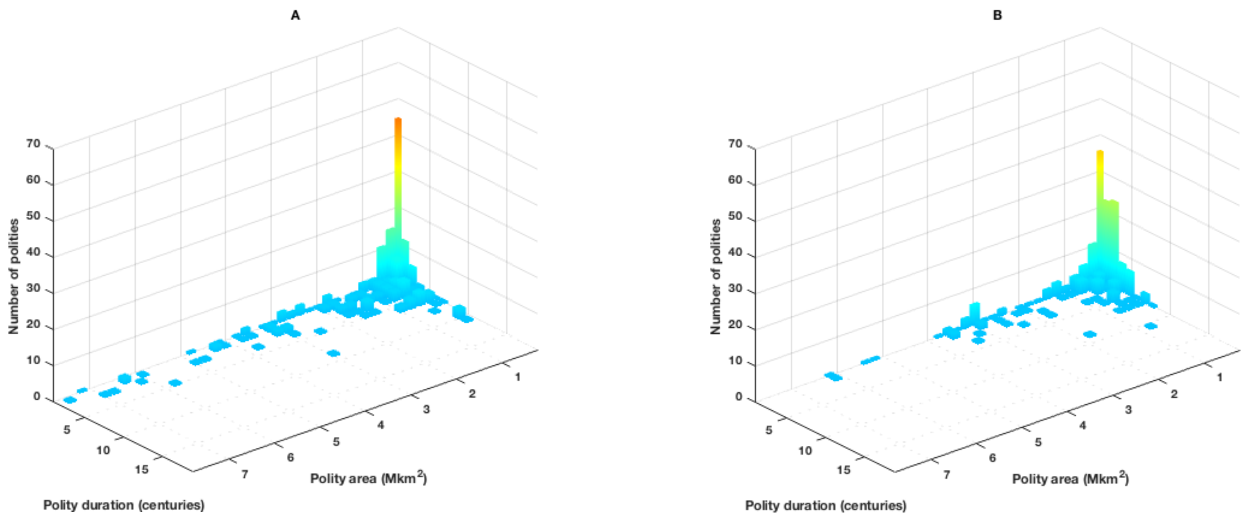

Fig S9: Historical (**A**) and mean predicted (**B**) distributions of peak (agrarian and nomadic) duration (in centuries) and peak size (in millions (M) of km^2) between 1500 BCE and 1500 CE assuming initial polities in the Ganges and Mekong river valleys. Compare predictions with Fig 5 in the main paper.

No delays in regional agricultural productivity

If all of the agricultural regions shown in Fig 3 of the main paper become as productive in 1500 BCE as they are by 1000 CE (and then double again in that year) the expansion of very large agrarian states after the nomadic threat arises in 1000 BCE is substantially enhanced. Indeed, nearly all the main Eurasian agricultural areas are occupied by states around 300 CE, 1200 years early (Fig S11). The population of Eurasia also increases with the precocious occupation of productive land, with the population apparently saturating the carrying capacity just after 500 CE, before the exogenous doubling in 1000 CE. The expansion of both Asian and European states are not constrained as they are when the Pearl river valley and north eastern Europe remain unproductive until 200 BCE and 300 CE, respectively.

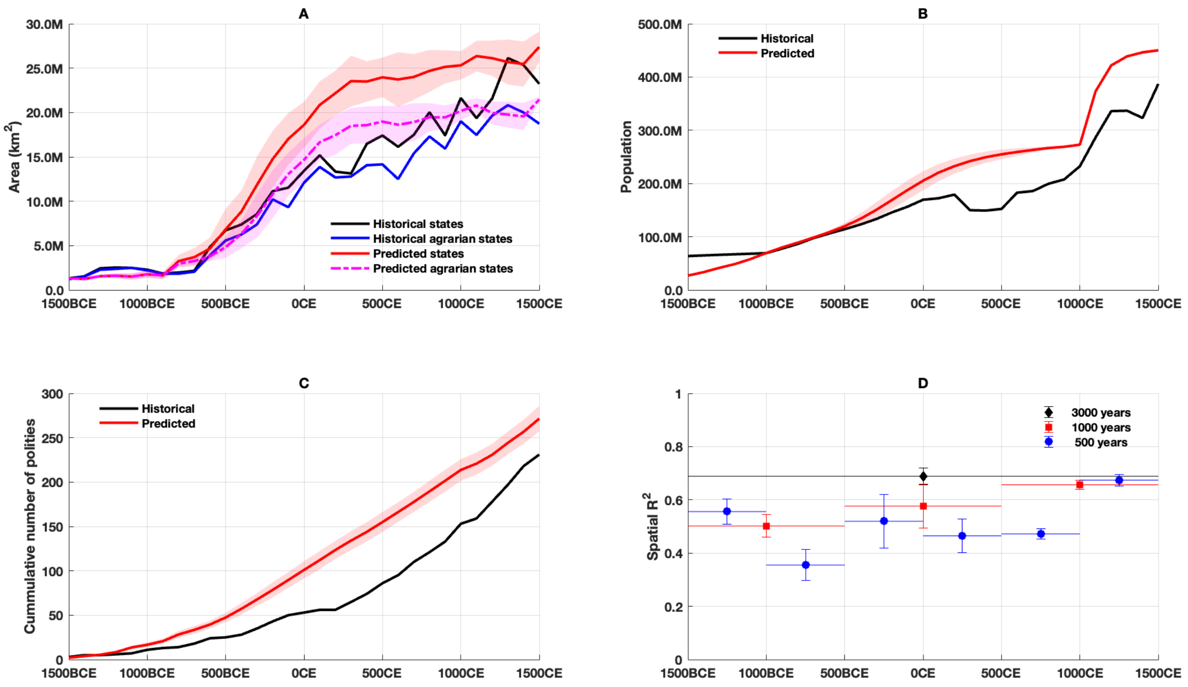

Fig S10: Predictions for the Old World without agricultural constraint based on 32 trials. **A**: Predicted mean and standard deviation of total area under all large-scale polities (red) and agrarian states (magenta) compared with historical values per century from TCTG13 (black and blue, respectively). **B**: Predicted mean and standard deviation of total population (red) compared with historical values every 20 years from KK10 (black). **C**: Predicted mean and standard deviation of cumulative (red) number of large-scale polities surviving a century compared with historical values (black) from the TCTG13 dataset. **D**: Mean and standard deviation of spatial R^2 metrics for 500 (blue), 1000 (red) and 3000 (black) year intervals. Compare predictions with Fig 4 in the main paper.

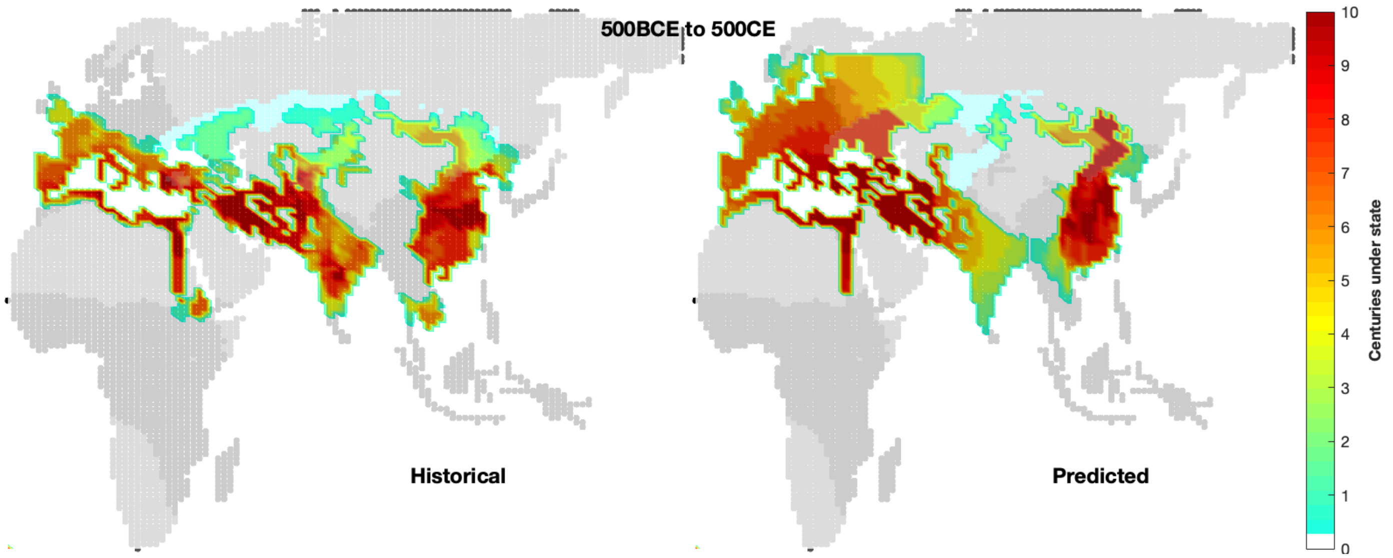

Fig S11: Mean large-scale polity density over 32 trials in the Old World for the 1000 years ending in 500 CE. Left panel reflects historical data from the TCTG13 dataset for large-scale states (≥ 10 regions); right panel reflects model predictions without agricultural constraint. Darker grey indicates all agricultural regions are available; light blue indicates steppe. Compare predictions with Fig 3B. Map, biome and historical data republished from [1] under a CC BY license, with permission from Proceedings of the National Academy of Sciences, original copyright 2013.

No steppe nomadic threat

In the absence of steppe nomadic threat (accomplished either by delaying its invention to 1500 CE or limiting the nomad's maximum s_n to match the initial agrarian state value) yet maintaining efficient sea and desert military travel, the agrarian states are never very powerful so they remain relatively small and are numerous (Fig S12C and Fig S14B). They are unable to occupy the entire Eurasian agricultural region in 3000 years; overall the spatial R^2 variance explained drops to 43%. The mean duration of states increases slightly (Fig S14B) and the proportion of states that succumb to demographic-structural crises increases to 48%, demonstrating that the nominal asymmetric and increased military efficiency triggered by the nomads cause more states to fall to invasion than would otherwise happen. Interestingly, Fig S12B indicates that in spite of diminished occupation of territory, the predicted Eurasian population remains comparable to historical and the nominal predictions. Note, however, that the population curve is just beginning to flatten after 500 CE (Fig S12B) indicating that the total agrarian state carrying capacity was saturating before the (exogenous) doubling of productivity in 1000 CE.

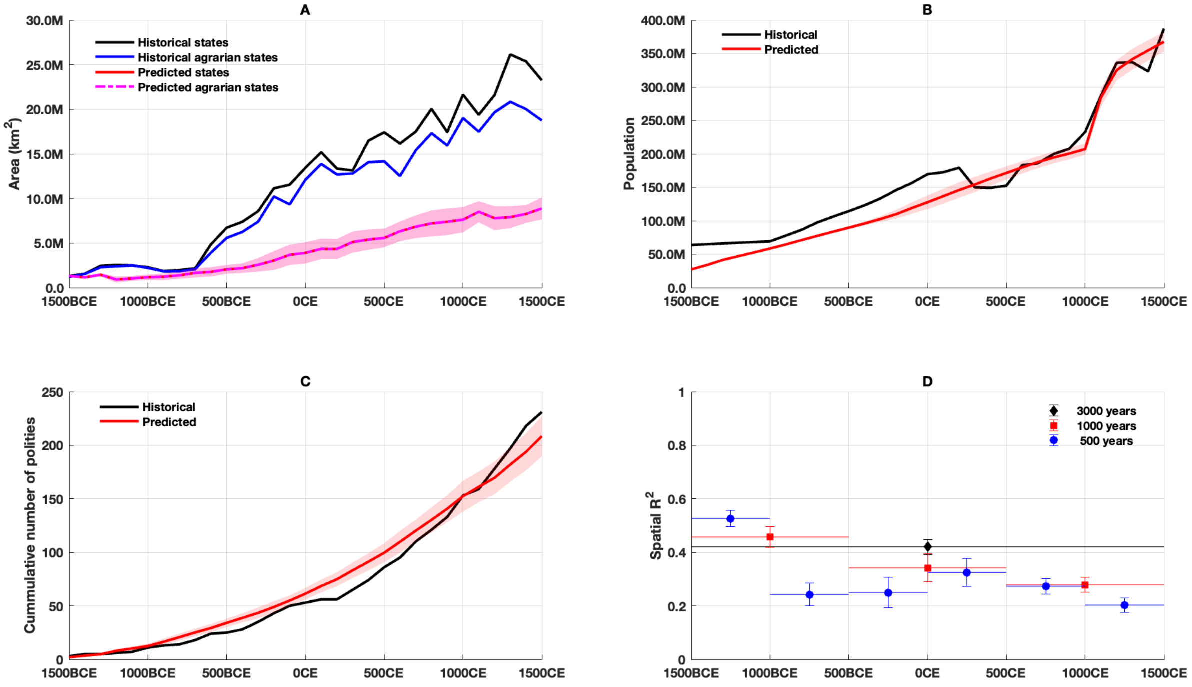

Fig S12: Predictions for the Old World without steppe nomadic threat based on 32 trials. **A**: Predicted mean and standard deviation of total area under all large-scale polities (red) and agrarian states (magenta) compared with historical values per century from TCTG13 (black and blue, respectively). **B**: Predicted mean and standard deviation of total population (red) compared with historical values every 20 years from KK10 (black). **C**: Predicted mean and standard deviation of cumulative (red) number of large-scale polities surviving a century compared with historical values (black) from the TCTG13 dataset. **D**: Mean and standard deviation of spatial R^2 metrics for 500 (blue), 1000 (red) and 3000 (black) year intervals. Compare predictions with Fig 4 in the main paper.

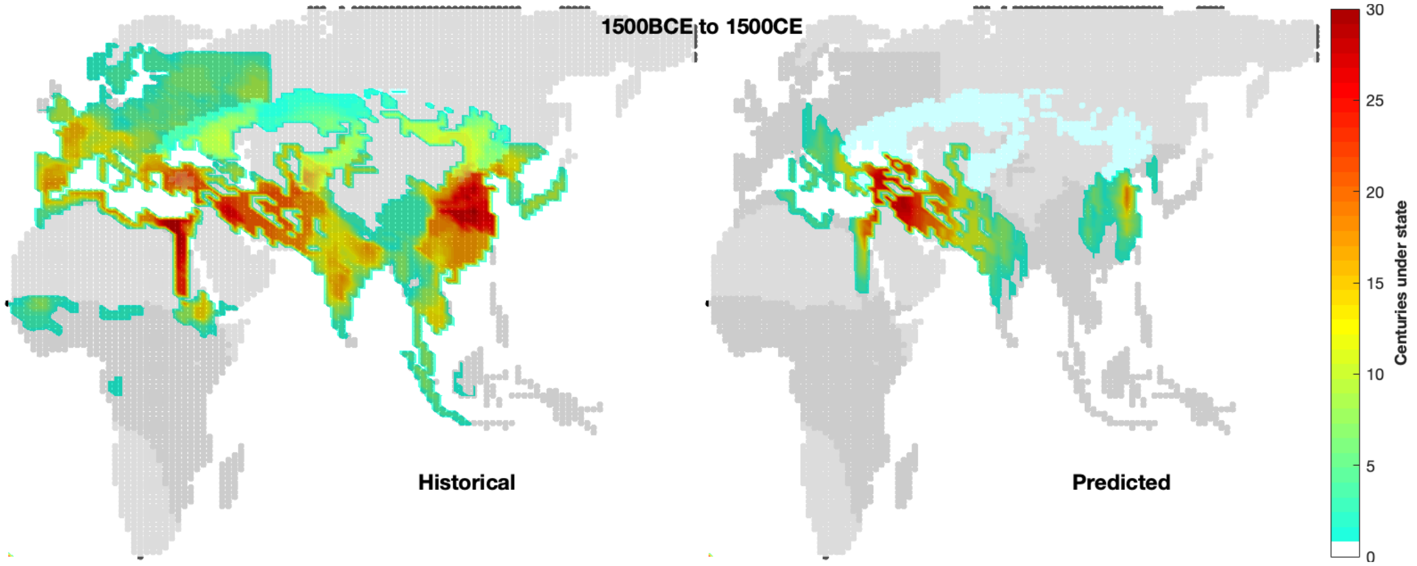

Fig S13: Mean large-scale polity density over 32 trials in the Old World for 3000 years beginning in 1500 BCE. Left panel reflects historical data from the TCTG13 dataset for large-scale states (≥ 10 regions); right panel reflects model predictions without steppe nomadic threat. Darker grey indicates available agricultural regions in the period; light blue indicates steppe. Compare predictions with Fig 3D. Map, biome and historical data republished from [1] under a CC BY license, with permission from Proceedings of the National Academy of Sciences, original copyright 2013.

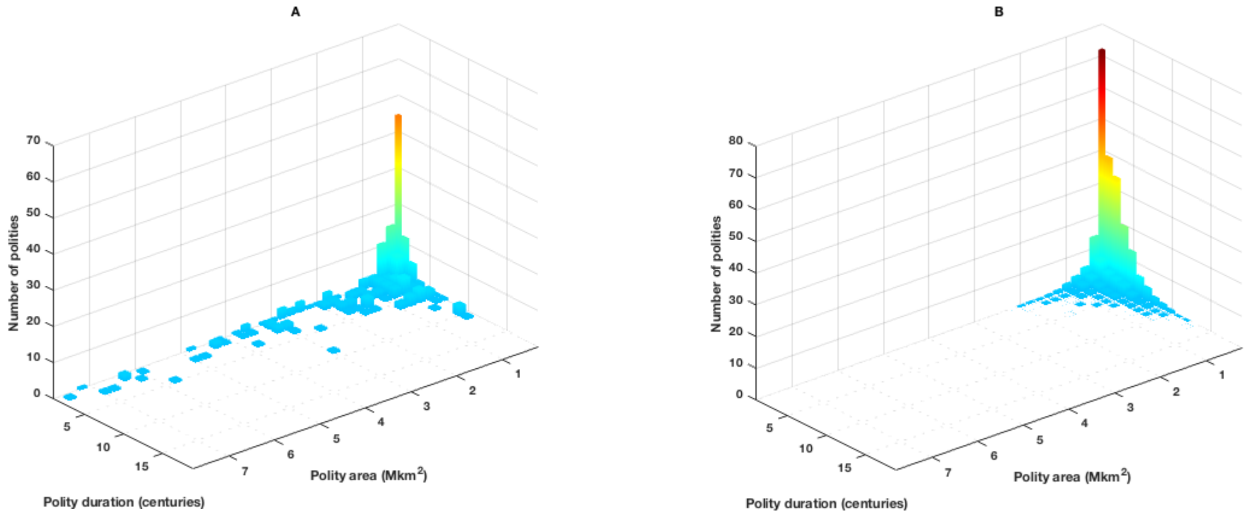

Fig S14: Historical (**A**) and mean predicted (**B**) distributions of peak (agrarian and nomadic) duration (in centuries) and peak size (in millions (M) of km^2) between 1500 BCE and 1500 CE without steppe nomadic threat. Compare predictions with Fig 5 in the main paper.

No steppe nomadic threat but agrarian states improve *s* ala Kremer, 1993

Kremer [13] posited (his Equation 3) that the change in a technology (A) was proportional to the total population (P) and the current level of technology:

$$\dot{A} = kPA \tag{10}$$

Assuming that military efficiency s reflects the only 'technology' being improved (i.e., $s = A$) and using the KK10 values for Old World P I estimate that $k \approx 1.3{\times}10^{-12}$ innovations/person/year permits s to double from 1.1. to 2.1 in 3000 years. Using this value, Fig S15 (right axis) shows the predicted s values at the end of a state's lifetime for a representative run compared to the KK10 estimated Old World population.

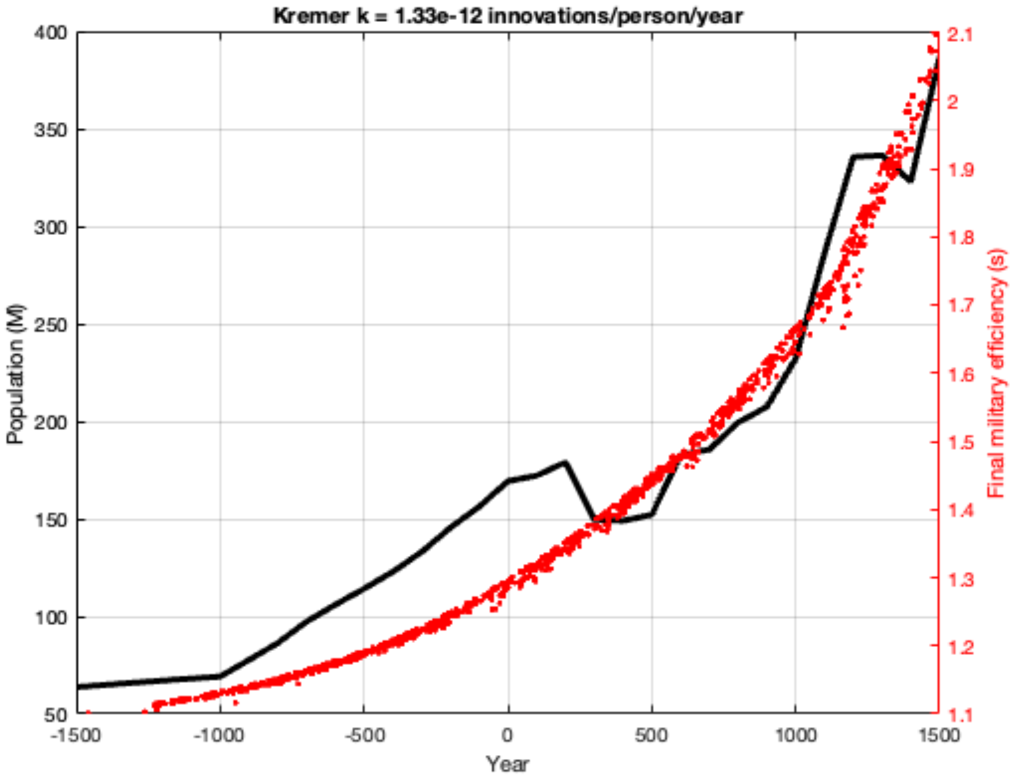

Fig S15: Achieved military efficiency (s, in red) based on Equation 10 and Old World population based on KK10 (black).

In these simulations, again, no nomadic threat occurs. Because the population is small at the start of the simulations, all states have equally small starting military efficiency and as a consequence they lag the expansion rate of historical states in the first 1000-year interval and, in spite of improvements, still lag somewhat in the second 1000-year interval. However, by the end of the third 1000-year interval states have occupied all the Eurasian agricultural regions. This lag-catch-up dynamic is shown in Fig S17A. Overall there are many more smaller states created (Fig S18B) and simulations indicate 40% of them fall to demographic-structural crises.

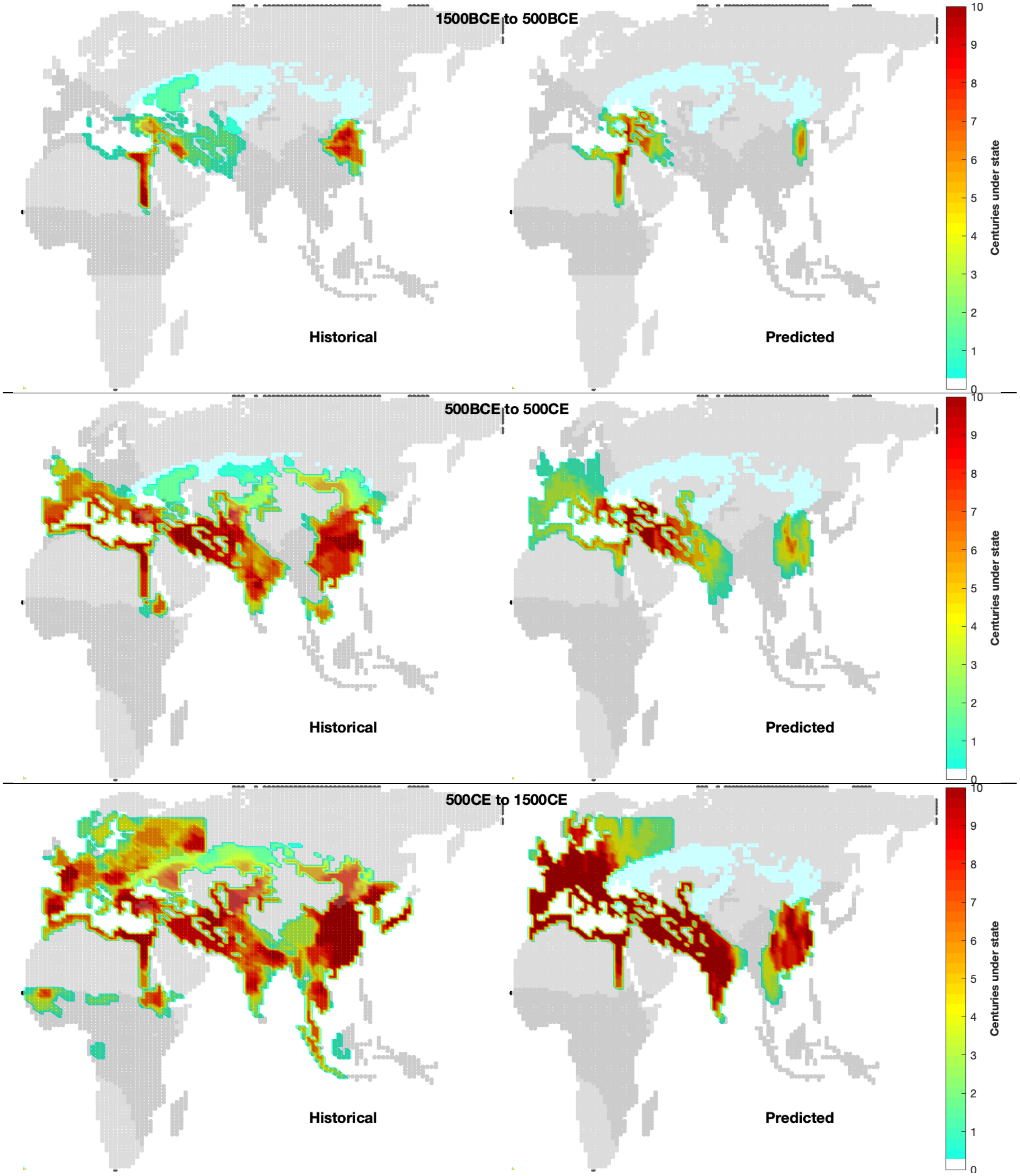

1500BCE to 500BCE
Historical
Predicted
500BCE to 500CE
Historical
Predicted
500CE to 1500CE
Historical
Predicted
Centuries under state
0
1
2
3
4
5
6
7
8
9
10

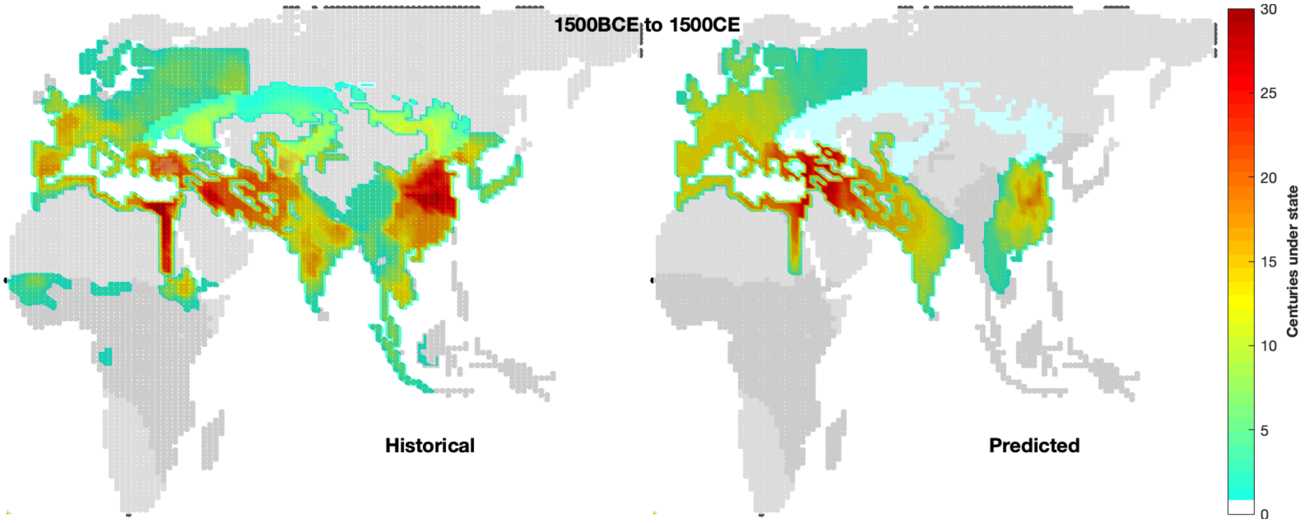

Fig S16: Mean large-scale polity density over 32 trials for the Old World at the end of each 1000-year interval (**A**, **B**, and **C**) and over 3000 years (**D**) beginning in 1500 BCE. Left panels reflect historical data from TCTG13 for large-scale states (≥ 10 regions); right panels reflect model predictions assuming no nomadic threat but endogenous military improvement ala [13]. Darker grey indicates available agricultural regions in each period; light blue indicates steppe. Compare predictions with Fig 3 in the main paper. Map, biome and historical data republished from [1] under a CC BY license, with permission from Proceedings of the National Academy of Sciences, original copyright 2013.

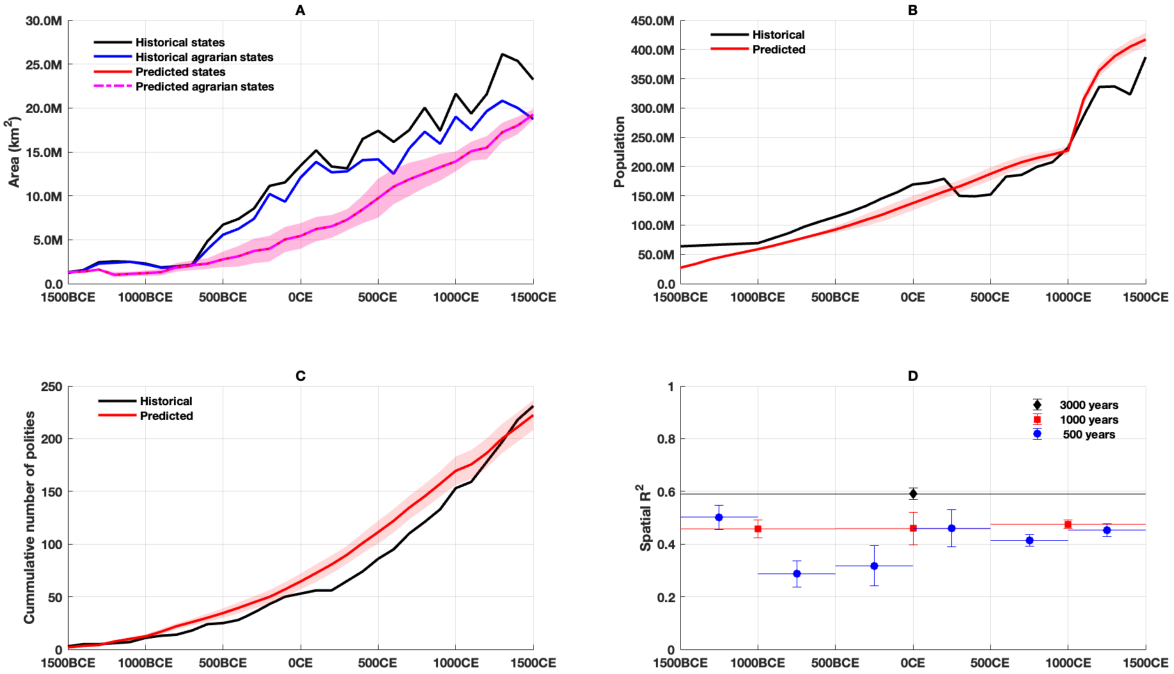

Fig S17: Predictions for the Old World assuming no nomadic threat but endogenous military improvement ala [13]based on 32 trials. **A**: Predicted mean and standard deviation of total area under all large-scale polities (red) and agrarian states (magenta) compared with historical values per century from TCTG13 (black and blue, respectively). **B**: Predicted mean and standard deviation of total population (red) compared with historical values every 20 years from KK10 (black). **C**: Predicted mean and standard deviation of cumulative (red) number of large-scale polities surviving a century compared with historical values (black) from the TCTG13 dataset. **D**: Mean and standard deviation of spatial R^2 metrics for 500 (blue), 1000 (red) and 3000 (black) year intervals. Compare predictions with Fig 4 in the main paper.

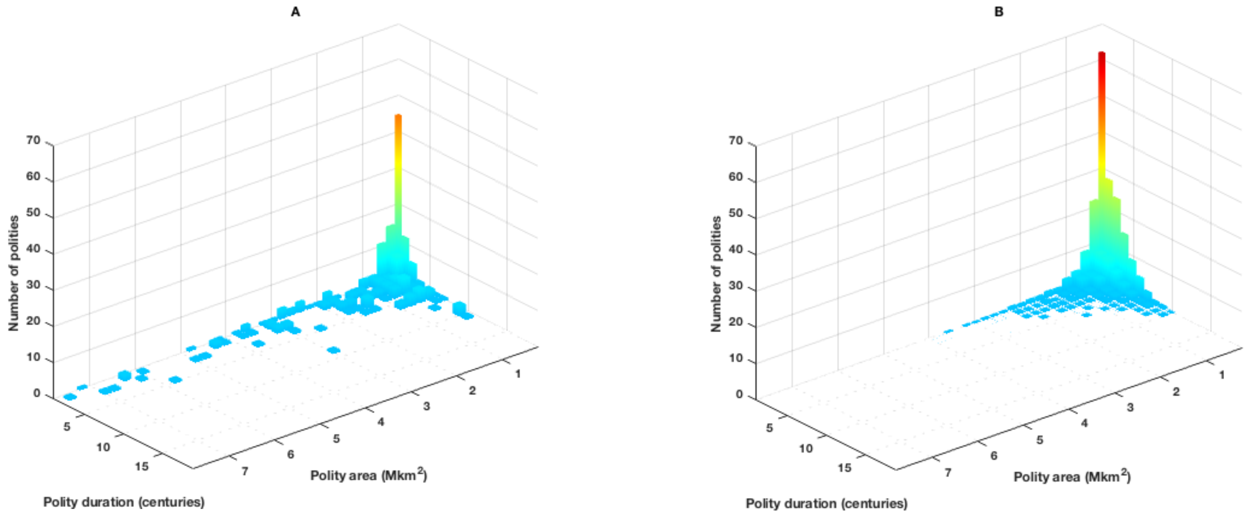

Fig S18: Historical (**A**) and mean predicted (**B**) distributions of peak (agrarian and nomadic) duration (in centuries) and peak size (in millions (M) of km^2) between 1500 BCE and 1500 CE assuming no nomadic threat but endogenous military improvement ala [13]. Compare predictions with Fig 5 in the main paper and Fig S14.

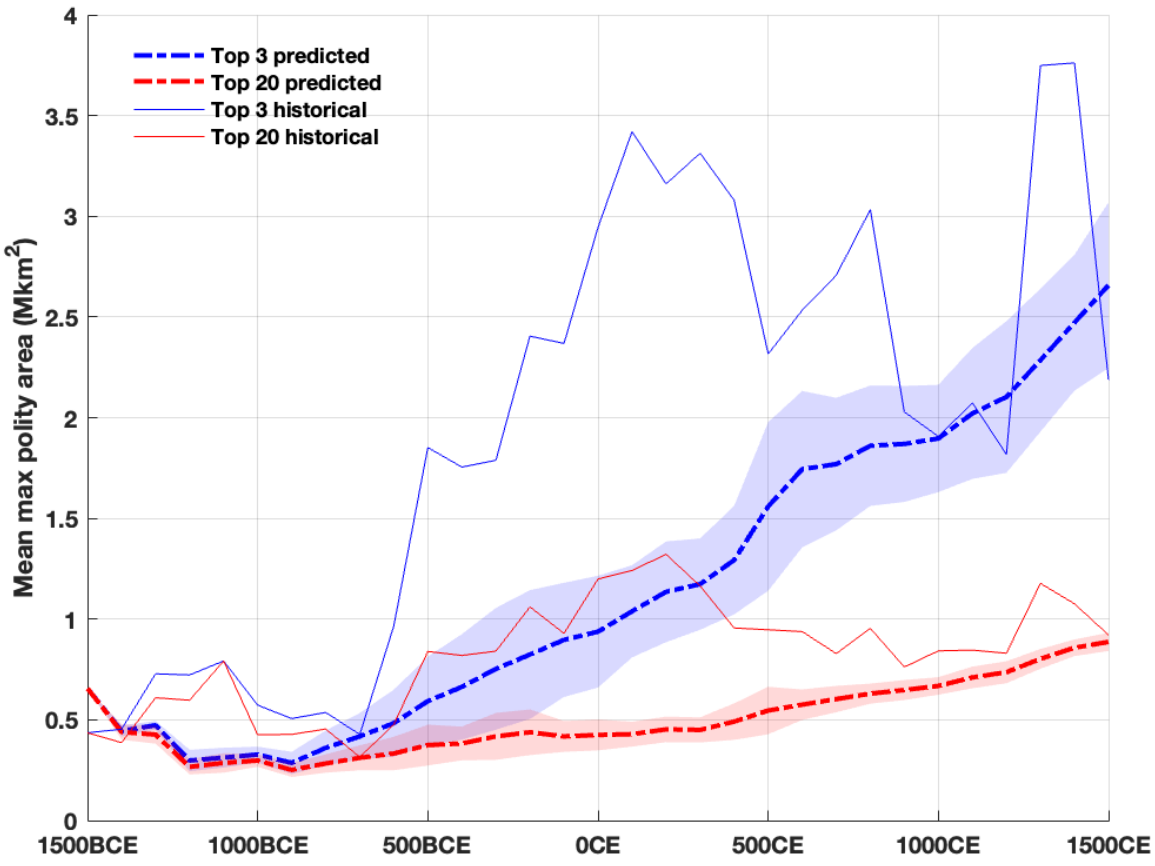

Fig S19: Mean and standard deviation of predicted versus historical polity sizes for the largest 3 and largest 20 states (agrarian and nomadic) between 1500 BCE and 1500 CE assuming no nomadic threat but endogenous military improvement ala Kremer, 1993. Compare predictions with Fig 6 in the main paper.

Inventing horse cavalry in east Asia in 1000 CE

Moving the invention of horse cavalry to the east Asian steppe border (around 112°E) in 1000 BCE causes the first Asian nomadic confederation and mirror agrarian state to appear around 500 BCE (Fig S20A). While slightly delayed, the dynamic replicates in the east the Achaemenid/Scythian dynamic in the west in the nominal simulation with nomadic tribes pestering Chinese states for 500 years while the diffusive delay heads in the reverse (westerly) direction. However, unlike east Asia, many more states arise in southwest Asia during the diffusion time because of the fragmented geography and the nearby spreading centers of the Fertile Crescent and the Nile Valley, preventing large states in that region. A delay in the rise of very large states in Europe and India occurs reducing the imperial density observed in those regions. Indeed, there is little concentration of states along the lip of the Mediterranean in Europe, spreading instead north into eastern Europe and northwestern Russia. In the south, states in India fail to meet the states expanding from the Khmer. Nevertheless, over the three thousand

years the overall statistics are largely the same, except for the expected poor spatial R^2 values between 1000 BCE and 500 BCE.

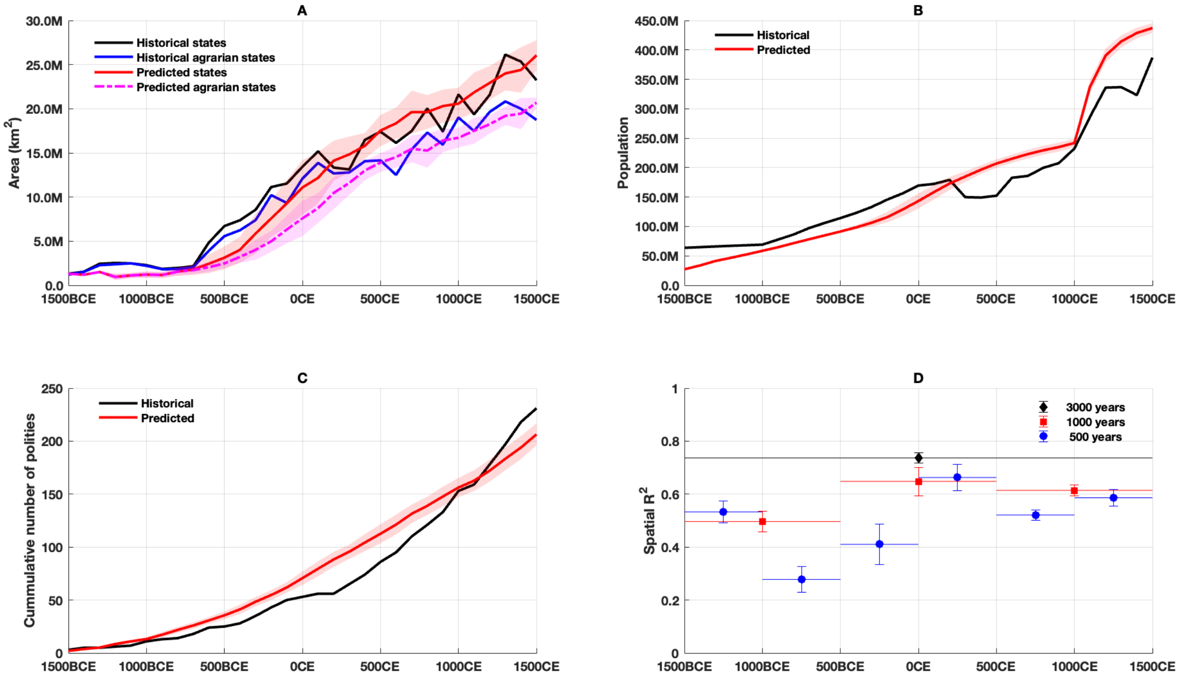

Fig S20: Predictions for the Old World assuming horse cavalry is invented in east Asia in 1000 BCE based on 32 trials. **A**: Predicted mean and standard deviation of total area under all large-scale polities (red) and agrarian states (magenta) compared with historical values per century from TCTG13 (black and blue, respectively). **B**: Predicted mean and standard deviation of total population (red) compared with historical values every 20 years from KK10 (black). **C**: Predicted mean and standard deviation of cumulative (red) number of large-scale polities surviving a century compared with historical values (black) from the TCTG13 dataset. **D**: Mean and standard deviation of spatial R^2 metrics for 500 (blue), 1000 (red) and 3000 (black) year intervals. Compare predictions with Fig 4 in the main paper.

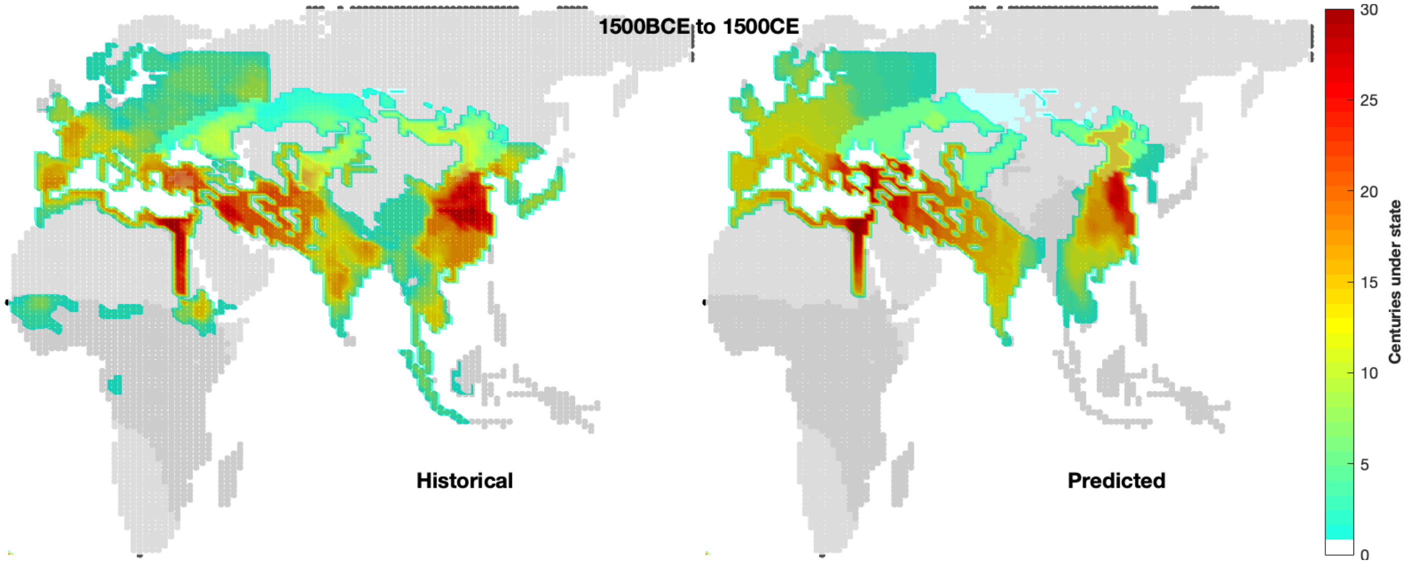

Fig S21: Mean large-scale polity density over 32 trials for the Old World for 3000 years beginning in 1500 BCE. Left panel reflects historical data from the TCTG13 dataset for large-scale states (≥ 10 regions); right panel reflects model predictions assuming horse cavalry is invented in east Asia in 1000 BCE. Darker grey indicates available agricultural regions in the period; light blue indicates steppe. Compare predictions with Fig 3D. Map, biome and historical data republished from [1] under a CC BY license, with permission from Proceedings of the National Academy of Sciences, original copyright 2013.

Inventing horse cavalry in response to agrarian population

Empirically I find the predicted population of the agrarian states within the Pontic-Caspian nomadic tribal strike zone in 1000 BCE (prior to extortion) to be roughly 3 million people. Triggering the invention of horse cavalry in any tribal strike zone in response to this level of extortable population, rather than by fiat in 1000 BCE, finds horse cavalry invention arising in the strike zone to the west of the Pontic Caspian steppe region around 1100 BCE (±100 years) (Fig S24). This western location, however, permits nomadic tribes there to pester the states in the Fertile Crescent and they develop rapidly both to the west into Europe via the Balkans and to the east into southwest Asia, as can be seen in the different imperial densities in both regions in Fig S23.

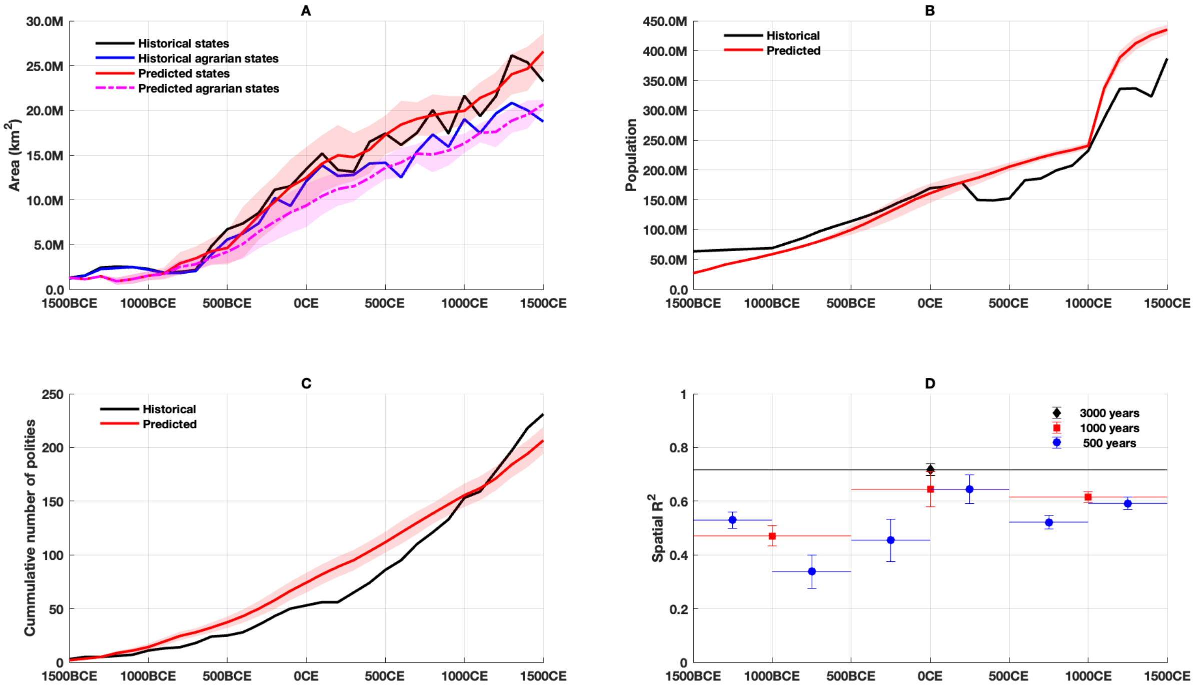

Fig S22: Predictions for the Old World assuming nomads invent horse cavalry whenever 3 million people appear within any nomadic tribal strike zone based on 32 trials. **A**: Predicted mean and standard deviation of total area under all large-scale polities (red) and agrarian states (magenta) compared with historical values per century from TCTG13 (black and blue, respectively). **B**: Predicted mean and standard deviation of total population (red) compared with historical values every 20 years from KK10 (black). **C**: Predicted mean and standard deviation of cumulative (red) number of large-scale polities surviving a century compared with historical values (black) from the TCTG13 dataset. **D**: Mean and standard deviation of spatial R^2 metrics for 500 (blue), 1000 (red) and 3000 (black) year intervals. Compare predictions with Fig 4 in the main paper.

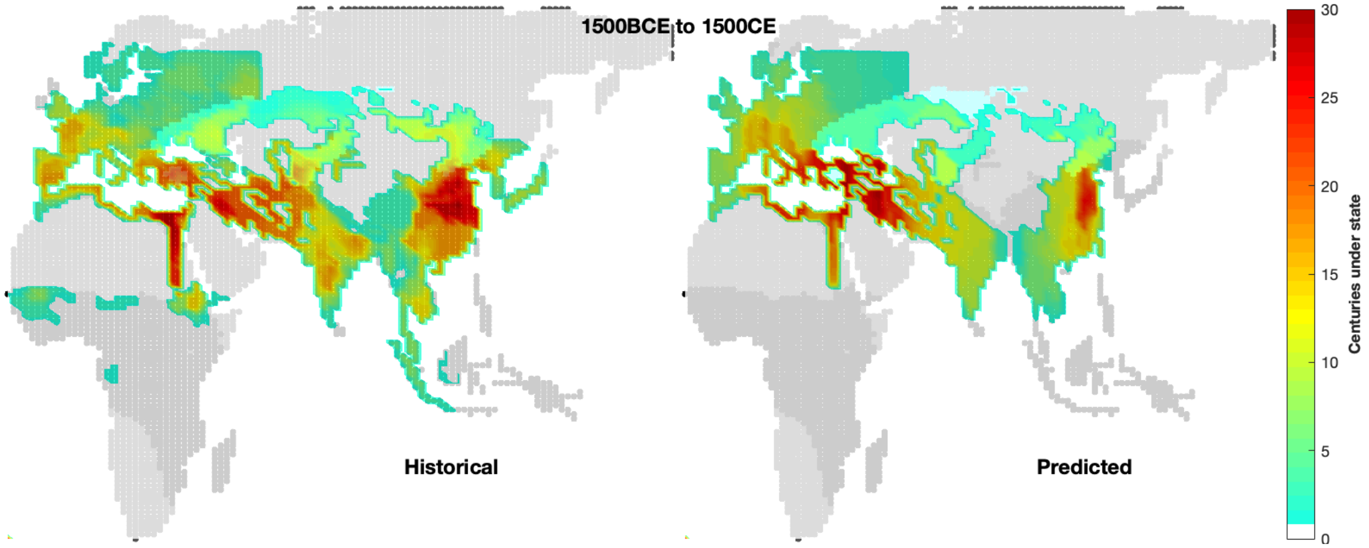

Fig S23: Mean large-scale polity density for the Old World for the 3000 years ending in 1500 CE. Left panel reflects historical data from the TCTG13 dataset for large-scale states (≥ 10 regions); right panel reflects model predictions assuming nomads invent horse cavalry whenever 3 million people appear within any nomadic tribal strike zone. Darker grey indicates available agricultural regions in the period; light blue indicates steppe. Compare predictions with Fig 3D in the main paper. Map, biome and historical data republished from [1] under a CC BY license, with permission from Proceedings of the National Academy of Sciences, original copyright 2013.

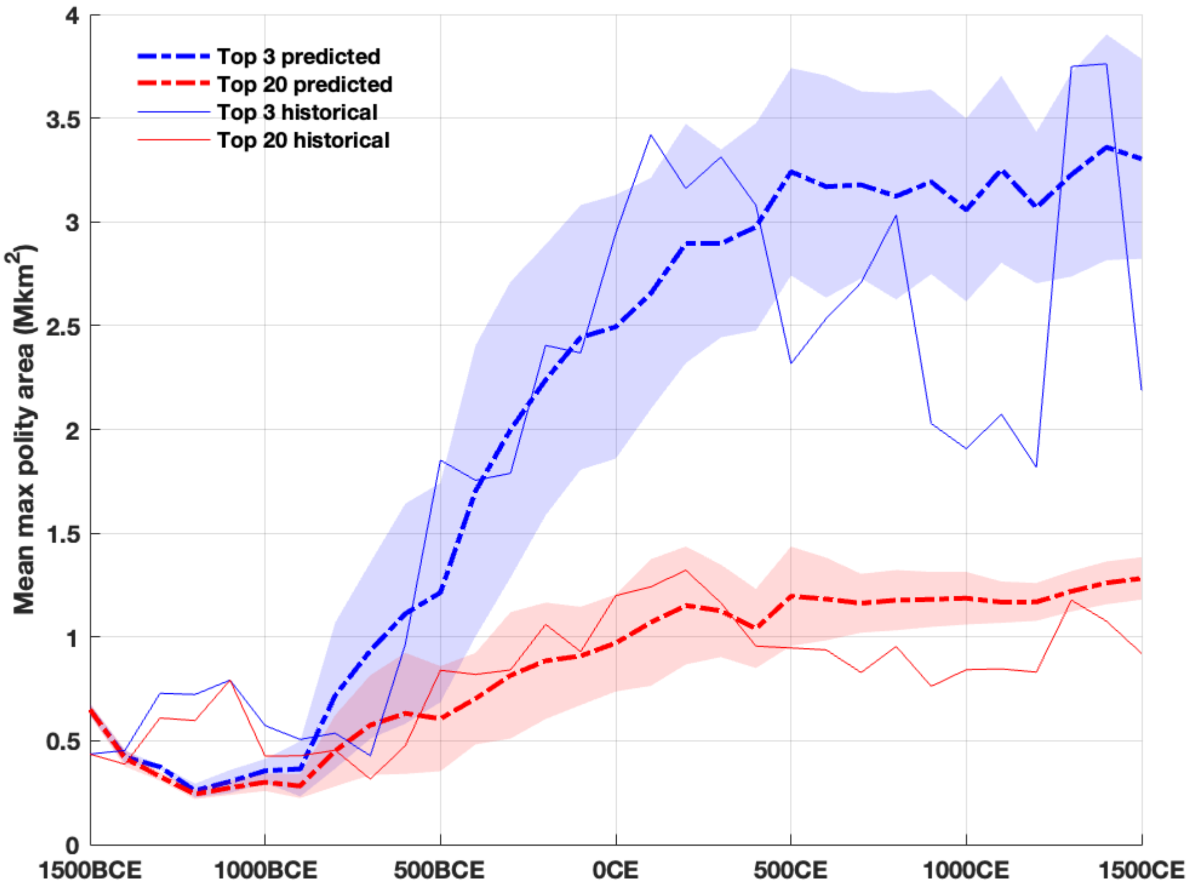

Fig S24: Mean and standard deviation of predicted versus historical polity sizes for the largest 3 and largest 20 states (agrarian and nomadic) between 1500 BCE and 1500 CE assuming nomads invent horse cavalry whenever 3 million people appear within any nomadic tribal strike zone. Compare predictions with Fig 6 in the main paper.

If the first large-scale states arose in east Asia in 1500 BCE their population achieves 3 million in the Asian strike zone around 1300 BCE with the first nomadic confederations arising there around 1000 BCE (Fig S27). Further, assuming the 500 year diffusion of horse cavalry to the west, confederations arise there around 400 BCE, delayed from the expected 800 BCE, as the population in the Near East must increase under nomadic tribal pressure (Fig S26).

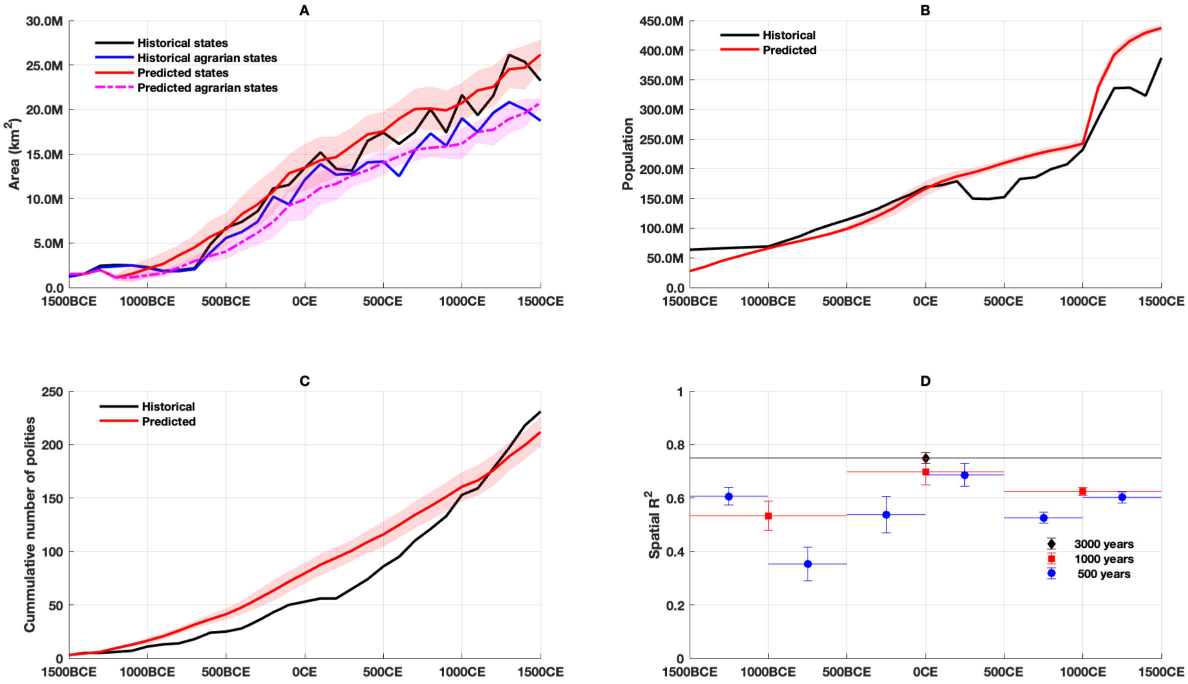

Fig S25: Predictions for the Old World assuming nomads invent horse cavalry whenever 3 million people appear within any nomadic tribal strike zone and Chinese states arise in 1500 BCE based on 32 trials. **A**: Predicted mean and standard deviation of total area under all large-scale polities (red) and agrarian states (magenta) compared with historical values per century from TCTG13 (black and blue, respectively). **B**: Predicted mean and standard deviation of total population (red) compared with historical values every 20 years from KK10 (black). **C**: Predicted mean and standard deviation of cumulative (red) number of large-scale polities surviving a century compared with historical values (black) from the TCTG13 dataset. **D**: Mean and standard deviation of spatial R^2 metrics for 500 (blue), 1000 (red) and 3000 (black) year intervals. Compare predictions with Fig 4 in the main paper.

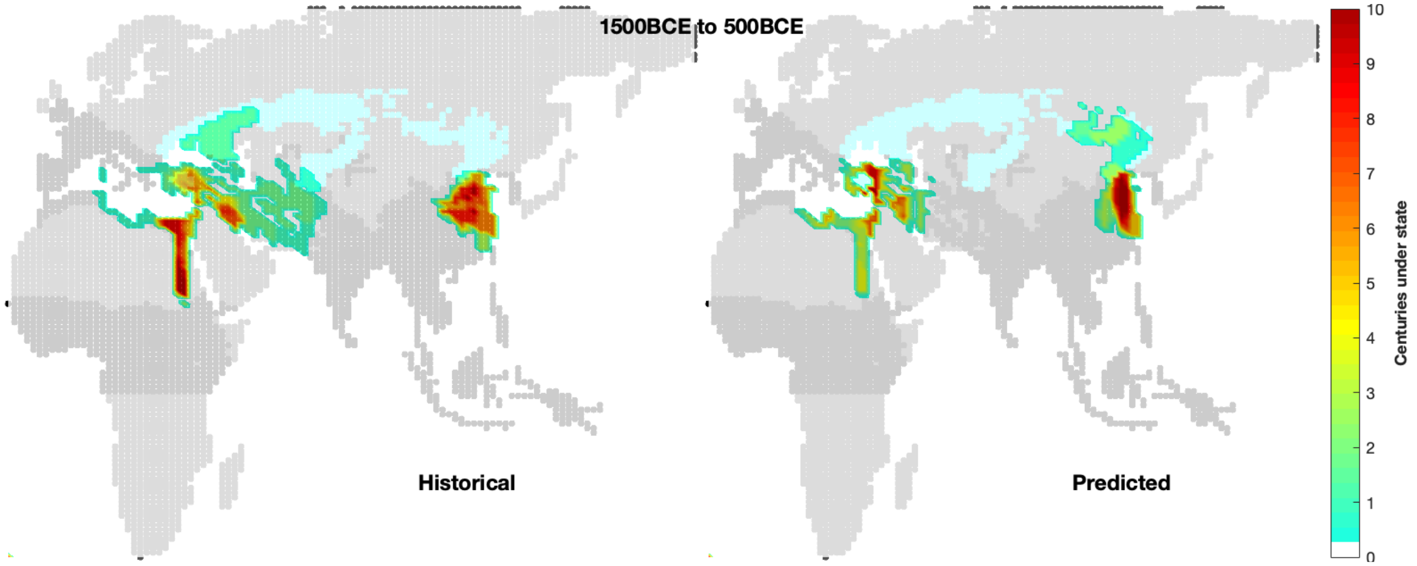

Fig S26: Mean large-scale polity density over 32 trials for the Old World for the 1000 years ending in 500 BCE. Left panel reflects historical data from the TCTG13 dataset for large-scale states (≥ 10 regions); right panel reflects model predictions assuming nomads invent horse cavalry whenever 3 million people appear within any nomadic tribal strike zone and Chinese states arise in 1500 BCE. Darker grey indicates available agricultural regions in the period; light blue indicates steppe. Compare predictions with Fig 3A in the main paper. Map, biome and historical data republished from [1] under a CC BY license, with permission from Proceedings of the National Academy of Sciences, original copyright 2013.

Higher Intra-state Elite Migration

Increasing the intra-state elite migration value (μ_ε) by an order of magnitude (1%/year) delays the time to reach a demographic-structural crisis and thus increases the mean time to state collapse to 2.4 centuries (from 1.7 centuries for the nominal simulations, a 40% increase). In addition the model predicts a slight decrease in number of states created and thus there are fewer shorter-lived states (Fig S27C and Fig S29B). Nevertheless, the fraction of states that eventually collapse from demographic-structural crises remains at 30%. While most other statistics remain similar the nominal simulation, there is a slight advance (with high variance) of the time of the first nomadic confederation (Fig S27A, just after 1000 BCE) and the expansion rate in southeast Asia is slowed so states there barely interact by 1500 CE (Fig S28B).

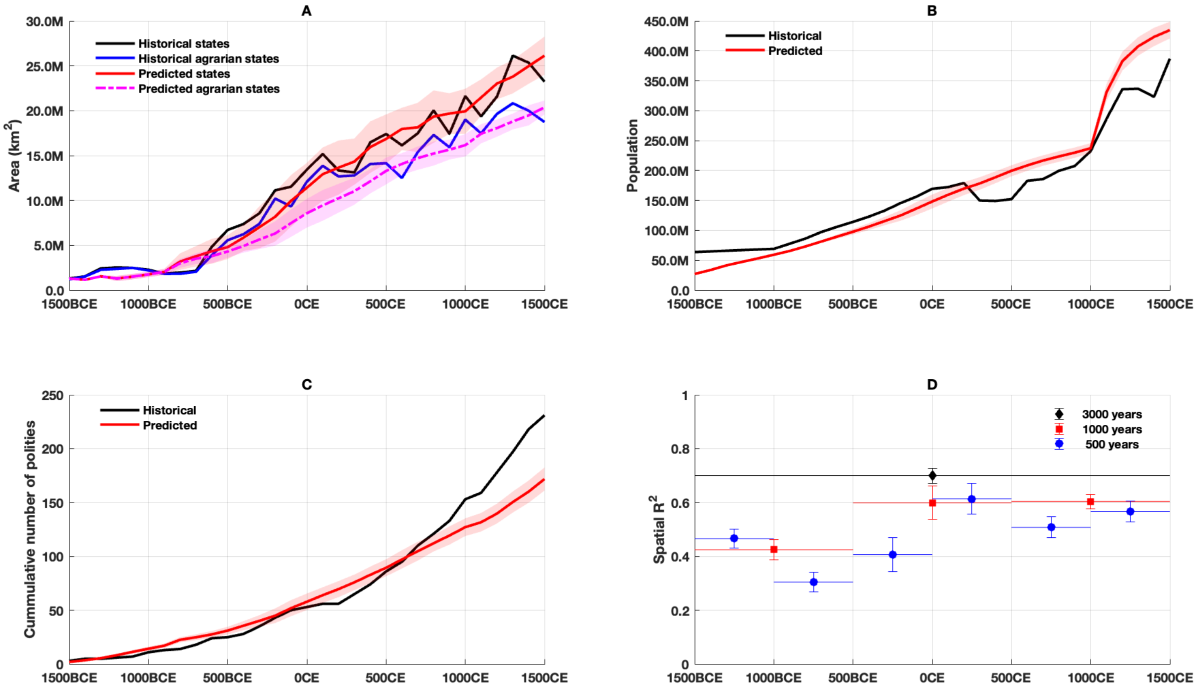

Fig S27: Predictions for the Old World assuming 1%/year elite migration based on 32 trials. **A**: Predicted mean and standard deviation of total area under all large-scale polities (red) and agrarian states (magenta) compared with historical values per century from TCTG13 (black and blue, respectively). **B**: Predicted mean and standard deviation of total population (red) compared with historical values every 20 years from KK10 (black). **C**: Predicted mean and standard deviation of cumulative (red) number of large-scale polities surviving a century compared with historical values (black) from the TCTG13 dataset. **D**: Mean and standard deviation of spatial R^2 metrics for 500 (blue), 1000 (red) and 3000 (black) year intervals. Compare predictions with Fig 4 in the main paper.

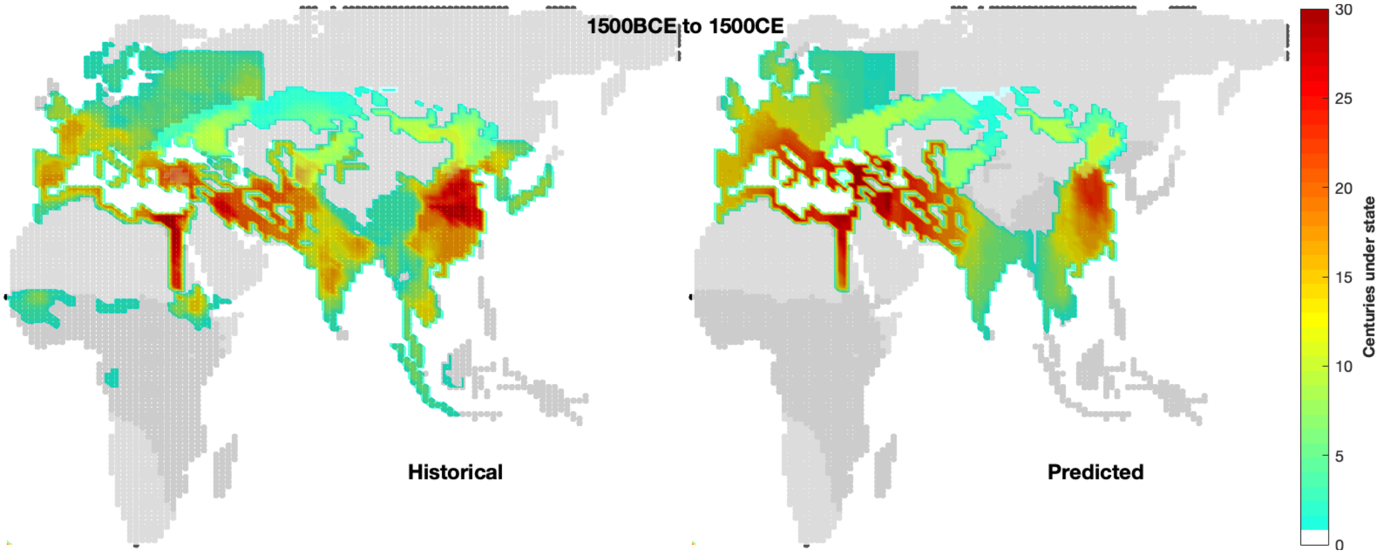

Fig S28: Mean large-scale polity density over 32 trials for the Old World for the 3000 years beginning in 1500 BCE. Left panel reflects historical data from the TCTG13 dataset for large-scale states (≥ 10 regions); right panel reflects model predictions assuming 1%/year elite migration. Darker grey indicates available agricultural regions in the period; light blue indicates steppe. Compare predictions with Fig 3D in the main paper. Map, biome and historical data republished from [1] under a CC BY license, with permission from Proceedings of the National Academy of Sciences, original copyright 2013.

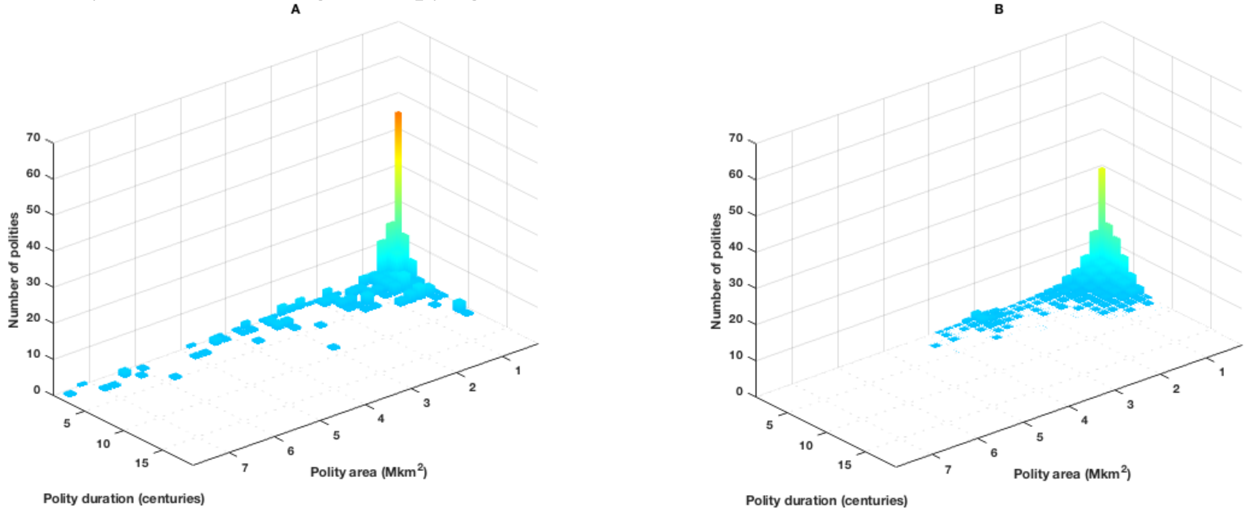

Fig S29: Historical (**A**) and mean predicted (**B**) distributions of peak (agrarian and nomadic) duration (in centuries) and peak size (in millions (M) of km^2) between 1500 BCE and 1500 CE assuming 1%/year elite migration. Compare predictions with Fig 5 in the main paper.

Bibliography

[1] Turchin P, Currie TE, Turner EAL, et al. War, space, and the evolution of Old World complex societies. *Proc Natl Acad Sci* 2013; 110: 16384–16389.

[2] Bennett JS. Repeated Demographic-Structural Crises Propel the Spread of Large-scale Agrarian States Throughout the Old World. *Cliodynamics* 2016; 7: 1–36.

[3] Kaplan JO, Krumhardt KM, Ellis EC, et al. Holocene carbon emissions as a result of anthropogenic land cover change. *The Holocene* 2011; 21: 775–791.

[4] Krumhardt KM. *ARVE Technical Report# 3: Methodology for worldwide population estimates: 1000 BC to 1850*. École Polytechnique Fédérale de Lausanne, Dept. of Environmental Engineering, ARVE Research Group., http://arve. epfl. ch/technical_reports/ARVE_tech_report3_pop_methods. pd (2010).

[5] McEvedy C, Jones R. *Atlas of world population history*. Penguin, 1978.

[6] Bennett JS. Modeling the large-scale demographic changes of the Old World. *Cliodynamics* 2015; 6: 57–76.

[7] Campbell BM. *English seigniorial agriculture, 1250-1450*. Cambridge University Press, 2000.

[8] Malthus TR. *Population: the first essay*. University of Michigan Press, 1798.

[9] Turchin P, Nefedov S. *Secular Cycles*. Princeton: Princeton University Press, 2009.

[10] Boulding KE. *Conflict and defense: A general theory*. New York: Harper Press, 1962.

[11] Goldstone JA. *Revolution and rebellion in early modern world*. Berkeley: University of California Press, 1991.

[12] Turchin P, Currie TE, Turner EAL. Mapping the Spread of Mounted Warfare. *Cliodynamics* 2016; 7: 217–227.

[13] Kremer M. Population growth and technological change: One million BC to 1990. *Q J Econ* 1993; 108: 681–716.
