# Supplementary material for: Retrodicting the rise, spread, and fall of large-scale states in the Old World
Source: PLoS One. 2022 Jan 12;17(1):e0261816. doi: 10.1371/journal.pone.0261816 (PMC8754308; doi:10.1371/journal.pone.0261816)
Supplement: Attachment — Submitted filename: Response_to_reviewers_Jun21.docx [file pone.0261816.s003.docx]

Responses to editor and reviewers in red below. I thank the editor and reviewers for their excellent, thoughtful comments and suggestions. I have followed their advice and moved the Materials and Methods section before Results and have changed the presentation of the Results to be more clear about the model results versus historical events. I have also improved the discussion of previous work, improved the figures, and attempted to reduce the redundancy in the document. I also revised the Supplemental Information to be consistent with the modifications in the main paper. Additional details about how I addressed specific points are included below. I hope this substantial revision addresses all concerns.

Journal Requirements:

When submitting your revision, we need you to address these additional requirements.

1. Please ensure that your manuscript meets PLOS ONE's style requirements, including those for file naming. The PLOS ONE style templates can be found at

<https://journals.plos.org/plosone/s/file?id=wjVg/PLOSOne_formatting_sample_main_body.pdf> and

<https://journals.plos.org/plosone/s/file?id=ba62/PLOSOne_formatting_sample_title_authors_affiliations.pdf>

The manuscript (and figure order) has been substantially changed to follow these formatting guidelines and Reviewer 2’s suggestion under their Format Point 2.

2. We note that Figures 1, 5, 6, 7, 9, S2, S4, S8, S11, S13, S16, S21, S23, S26, S28 and S2 Animation in your submission contain map images which may be copyrighted. All PLOS content is published under the Creative Commons Attribution License (CC BY 4.0), which means that the manuscript, images, and Supporting Information files will be freely available online, and any third party is permitted to access, download, copy, distribute, and use these materials in any way, even commercially, with proper attribution. For these reasons, we cannot publish previously copyrighted maps or satellite images created using proprietary data, such as Google software (Google Maps, Street View, and Earth). For more information, see our copyright guidelines: <http://journals.plos.org/plosone/s/licenses-and-copyright>.

These images (and movies) are generated by my analysis software based on the underlying latitude and longitude data provided by Turchin, et al. 2013 referenced in the paper. There are no copyright issues with these original images.

Reviewers' comments:

Reviewer's Responses to Questions

**Comments to the Author**

1. Is the manuscript technically sound, and do the data support the conclusions?

The manuscript must describe a technically sound piece of scientific research with data that supports the conclusions. Experiments must have been conducted rigorously, with appropriate controls, replication, and sample sizes. The conclusions must be drawn appropriately based on the data presented.

Reviewer #1: Yes

Reviewer #2: Partly

2. Has the statistical analysis been performed appropriately and rigorously?

Reviewer #1: Yes

Reviewer #2: Yes

3. Have the authors made all data underlying the findings in their manuscript fully available?

The [PLOS Data policy](http://www.plosone.org/static/policies.action#sharing) requires authors to make all data underlying the findings described in their manuscript fully available without restriction, with rare exception (please refer to the Data Availability Statement in the manuscript PDF file). The data should be provided as part of the manuscript or its supporting information, or deposited to a public repository. For example, in addition to summary statistics, the data points behind means, medians and variance measures should be available. If there are restrictions on publicly sharing data—e.g. participant privacy or use of data from a third party—those must be specified.

Reviewer #1: Yes

Reviewer #2: Yes

4. Is the manuscript presented in an intelligible fashion and written in standard English?

PLOS ONE does not copyedit accepted manuscripts, so the language in submitted articles must be clear, correct, and unambiguous. Any typographical or grammatical errors should be corrected at revision, so please note any specific errors here.

Reviewer #1: Yes

Reviewer #2: Yes

5. Review Comments to the Author

Please use the space provided to explain your answers to the questions above. You may also include additional comments for the author, including concerns about dual publication, research ethics, or publication ethics. (Please upload your review as an attachment if it exceeds 20,000 characters)

Reviewer #1: This paper represents a significant step towards understanding the limited set of factors driving large-scale repetitive patterns in the long-term socio-political development in the “Old World”. Model outcomes correspond to a significant number of values obtained from historical data. The disagreements between modeled and historical data are discussed in the text. Model assumptions are clearly presented. I suggest just a few minor improvements clarifying the text and slightly extending the discussion of the model outcomes. These are as follows:
1. Discussing the increase in military efficiency of steppe populations c. 1000 BCE (vs. 1500 BCE) it might be reasonable to specify that nomadisim as a form of economic organization and the lifeway in steppe forms in the western part of the Great Eurasian Steppe in the very end of the 2nd mil BCE – the very beginning of the 1st mil. BCE (e.g., Khazanov 1984; Bunyatyan 2003). Leaving aside the issues of earlier origin of horse riding and military conflicts between Eneolithic and Bronze Age semi-nomads and agriculturalists (e.g. Anthony 2007), Khazanov’s conclusion that the Bronze Age semi-nomads did not seem to be a real threat to Early States supports the model’s assumptions (pp. 4-5) and outcomes. Khazanov’s book also discusses the appearance of nomads in the eastern part of the Great Eurasian Steppe c. in the middle of the 1st mil BCE, which finds an agreement with the results of simulations.

The initial paragraph under Nomadic Tribes and Confederations has been improved to reflect the change in political and economic organizations discussed by Khazanov.

2. From the perspective of archaeological record, localization of the origin of “military cavalry” between the Black Sea and the Caspian Sea raises doubts. Synchronous horse riding and arrowheads, short swords and daggers are evident across much wider territory – from the North Pontic region to the Volga region. Maybe, it would be more reasonable to emphasize the role of military tactics already assumed by the author. This would provide a stronger ground for localization of the invention and formation of the nomads federation in the smaller region.

An excellent point. I’ve changed the text to read Pontic Caspian steppe region and changed Figure 2A (previous Figure 9A) to outline that entire area, which includes the drainage of the Volga.

3. Sarmatian time (c. 4th/3rd century BCE – 4th century BCE) in the western part of the Great Eurasian Steppe is associated with the invention of stirrup causing the appearance of heavily equipped riders known in this area from the 1st century BCE (e.g., Simonenko 2015 and earlier papers by Khazanov). Huns (4th – 6th century BCE) were equipped by complex compound bows. I would briefly discuss how these military inventions in steppe fit the model outcomes.

I added a paragraph at the end of the Nomadic Tribes and Confederations section to address this observation.

4. It seems to be reasonable to add a few sentences summarizing the outcomes of Bennett 2016 paper in the introduction (p. 5, line 94). Otherwise, reading may be somewhat biased from all the model assumptions and parameters towards the arm race between two types of polities.

Introduction text has been improved to summarize the Bennett 2016 outcomes and compare and contrast them with Turchin et. al.’s 2013 model.

5. Since the model starts at 1500 BCE, maybe it would be reasonable to replace the “Middle Bronze Age” by the “Late Bronze Age” according to the Bronze Age periodization developed for the Near East and the western part of the Great Eurasian Steppe? I am not familiar with the periodization of the eastern part of steppe – if this date corresponds to the Middle Bronze Age there, maybe “Middle/Late Bronze Age”?

Agreed; the text throughout has been changed to ‘late Bronze Age’.

6. Figure 4 in the text is referenced before Figure 1 (p. 3, line 64). I would remove this reference in order to keep the ordering of figures and references to them.

Figure references changed with the manuscript rearrangement and are now in sequential order.

7. P. 17, line 373, “only” – please replace the semicolon by “l”.

Fixed.

Anthony, D.W. (2007). The Horse, the Wheel and Language. How Bronze-Age Riders from Eurasian Steppe Shaped the Modern World. Princeton: Princeton University Press.
Bunyatyan, K.P. (2003). Correlations between agriculture and pastoralism in the Northern Pontic Steppe area during the Bronze Age. In: Levine, M., Renfrew, C. and Boyle, K. (eds.), Prehistoric Steppe Adaptation and the Horse. Cambridge: McDonald Institute for Archaeological Research, 269-286.
Khazanov, A. (1984). Nomads and the Outside World. Cambridge: Cambridge University Press.
Simonenko, A. (2015). Sarmatskie vsadniki Severnogo Prichenomorya [2nd edition]. Kiev: Oleh Filiuk. [English transliteration of the Russian title].

Reviewer #2: ## Summary and recommendation

This work attempts to retrodict the trajectories of large states in Afro-Eurasia between 1500 BCE and 1500 CE, building over the work of Turchin et al. on macro-scale modeling. The author presents a new version of Turchin et al. model where there are extra contingencies to specific mechanisms and, most significantly, where steppe nomadic confederations are implemented differently from agrarian states.

The paper is focused on presenting, interpreting, and discussing simulation results, while essential elements of model description are somewhat lacking or only found in Supplementary Information files. Unfortunately, the manuscript is also excessively long and not well-structured, which delays proper comprehension and assessment. The author has invested significant work in developing the model, running experiments, and analyzing results; however, the delivery of contents, including text and figures, and its contextualization within historical investigation does not make justice to this effort.

I believe this work is worth publishing and that it has the potential to be an important contribution, relevant to a broad audience. However, I recommend its publication only after addressing the following points.

\## Comments on Manuscript

*Content*.

Point 1. The manuscript covers several aspects that either follow, extend, or revise Turchin et al. and their sources. The author is clearly aligned with a general model of Eurasian "cliodynamics" based on a clear-cut separation (and political and military opposition) between so-called agricultural and nomadic polities. This position is entirely valid. However, the author should at least mention (and potentially discuss) a very different perspective, quite extended among historians and archaeologists. This other line of thought considers that the degree and nature of this separation have been exaggerated by biased discourses, encouraged by contemporary and later polities, up to modern nation-states. I highly recommend taking into consideration the work of Anatoli Khazanov (e.g., his inaugural book, "Nomads and the Outside World", 1984). More generally, the manuscript would benefit from acknowledging the nuances indicated by many historians and archaeologists working in Afro-Eurasia. For example, specifically on Central Asia, see Michael Frachetti's work on the so-called Inner Asia Corridor, Christoph Baumer's book "The History of Central Asia", and UNESCO's "History of Civilizations of Central Asia".

- Frachetti MD, Smith CE, Traub CM, Williams T. 2017 Nomadic ecology shaped the highland geography of Asia's Silk Roads. Nature 543, 193–198. (doi:10.1038/nature21696)

- Baumer C. 2012 The History of Central Asia: Volume 1: The Age of the Steppe Warriors. I. B. Tauris.

- Asimov MS, Bosworth CE, editors. 1998 History of Civilizations of Central Asia, Volume IV, The age of achievement: A.D. 750 to the end of the fifteenth century, Part One: The historical, social and economic setting. UNESCO Publishing. (the entire collection can be found here: <https://en.unesco.org/themes/generalregionalhistories#centralasia>)

I expanded the discussion about the model’s simple military assumptions in the Discussion, acknowledging that they ignore the complex and nuanced behaviors of *both* types of states.

Point 2. There is a general over-explanation of results in qualitative terms while lacking details about quantitative patterns and the more systematic comparisons between simulations and in relation to the historical data. The tone in Results dangerously confounds simulations and history, adding too much ad hoc interpretation and skipping important model details, which are left somewhat disconnected in S1, which in turn mixes model description and results. Simulation run "chronicles" can be helpful to illustrate the model common behaviors and even raise insight but are a limited resource in terms of drawing theoretical generalizations. I understand the challenge of dealing with simulations that take up to 10 minutes and output large amounts of spatially distributed data. Still, fitting results to the historical data must come together with a clear explanation of how the mechanisms programmed affect aggregated results, which is partially already done in S1 but not so much in the main text.

I have rewritten the Results discussion to better describe and demark the emergent simulation behavior and to separate how the simulation results compare to historical events. In particular I avoid using suggestive historic state names to identify simulated behavior and timing and instead use geographical region names.

*Format*.

Point 1. The paper is fundamentally clear but excessively long and wordy, sometimes even repetitive. Respecting the author's style, I would recommend at least re-visiting the general structure of the text and re-assessing what is explained in each paragraph and how (e.g., summarizing them as "titles" and checking if their content connects well with the rest of the flow).

I have attempted to address these concerns with the manuscript rearrangement and some rewriting to reduce redundancy.

Point 2. As I understand, PLOS ONE format does not place Materials and Methods at the end of research papers, so I highly recommend this section to be moved to its proper place after Introduction. This note is vital for an article dealing with a simulation model. In this case, the "methods" are not protocols applied to empirical data in order to obtain results (i.e., a mean to an end) but contains in itself the core of the contribution, which is mainly a theoretical and methodological proposition. For readers, it is not possible to evaluate results or even fully understand their implications if a minimum overview of the model does not precede them.

The manuscript (and figure order) has been changed to follow this suggestion.

Point 3. There are few and scattered mentions of model assumptions and "hard-coded" behaviors. I strongly suggest adding a bullet-point list, table, or diagram showing the contingencies that are pre-programmed by "deus_ex_machina()".

The nominal exogenous contingent behaviors are now summarized as a bullet list in Supplemental Information, along with the tables of nominal parameters.

Point 4. Also, the manuscript could benefit from re-evaluating the role of figures and their order. I strongly recommend that figures are numbered following their mention in the text as I believe it is a general editorial policy. For instance, Fig.4 is the first figure mentioned already in the Introduction. Is this mention necessary? If so, would it not be a better idea to represent the historical data considered in a way that is clearly differentiated from simulation results (as a new Fig. 1)?

The figure order has changed with the manuscript reorganization and reflects this suggestion.

Point 5. Regarding the main form of visualization of results (maps in Fig. 1, 5, 6, etc.), I recommend that notations and the meaning of colors be shown directly on the figure (i.e., titles, legend, etc.), particularly in the case of Fig. 1.

I added a color legend to each panel of the initial imperial density results figure in the main paper (new Fig 3, prior Fig 1). In order to reduce clutter I indicate in the caption of new Fig 8 (prior Fig 6) of the main paper that the scale is the same as Fig 3D. I updated all the imperial density figures in Supplemental Information to include the color legend.

Point 6. Figures 8 and 10 are too abstract and crude and do not improve the reader's understanding of the model. I would recommend searching for alternative types of diagrams and infographics, including at least some text in the figure.

With the manuscript rearrangement, I concur with the reviewer that they add little to understanding the model. I removed both figures.

---
\## Comments on Code and code documentation

Please publish the content of "S3 Code.zip" as a repository, following the FAIR principles, including making it citable. This is paramount for upholding the value of the model and the article in the long run. Offering software as Supplementary Information to the article is not a good option in general, but here it is particularly unacceptable given the importance, size, and complexity of the content. I would personally also include S1 and S2 to S3 together in a separate public and published repository. The materials can then be cited using DOI referring to stable repository servers.

Done. See https://doi.org/10.5281/zenodo.5020233

I recognize the effort to explain the code in "README.md", but I would strongly recommend following a more visual and structured approach (i.e., sections and subsections, bullet points, tables, figures). Furthermore, I believe much of what is explained in S1 should be present here as the model documentation, including some direct demonstrations of the mechanisms implemented in each method. In any case, this should not be kept in S1, which passed a few years may well become inaccessible or unfindable.

As an expedient I added the SI pdf to the repository above. Developing the visual or structured documentation, which I have attempted in the past but with little success, will take more time than allotted for this review response.

I ask the author to be more precise when naming methods and variables and closely follow the single responsibility principle. The current names are amusing and understandable enough after further investigation. Still, the entire code would become easier to read and understand if the relationship between name and content/meaning would be more precise. Most methods are also quite long and complex (e.g., deus_ex_machina()) and could benefit from being broken down into smaller methods (i.e., refactoring).

I deeply thank the reviewer for reading the code and documentation. It definitely suffers from solo development over several years and could do with a good code review. I did refactor deus_ex_machina() and setup_for_trial() in NADSM which was straightforward enough. I welcome other specific suggestions.

6. PLOS authors have the option to publish the peer review history of their article ([what does this mean?](https://journals.plos.org/plosone/s/editorial-and-peer-review-process#loc-peer-review-history)). If published, this will include your full peer review and any attached files.


If you choose “no”, your identity will remain anonymous but your review may still be made public.


**Do you want your identity to be public for this peer review?** For information about this choice, including consent withdrawal, please see our [Privacy Policy](https://www.plos.org/privacy-policy).

Reviewer #1: No

Reviewer #2: **Yes:** Andreas Angourakis
