# Supplementary material for: Retrodicting the rise, spread, and fall of large-scale states in the Old World
Source: PLoS One. 2022 Jan 12;17(1):e0261816. doi: 10.1371/journal.pone.0261816 (PMC8754308; doi:10.1371/journal.pone.0261816)
Supplement: Attachment — Submitted filename: Response_to_reviewers_Nov21.docx [file pone.0261816.s004.docx]

Responses to editor and reviewers in red below.

Journal Requirements:

Please review your reference list to ensure that it is complete and correct. If you have cited papers that have been retracted, please include the rationale for doing so in the manuscript text, or remove these references and replace them with relevant current references. Any changes to the reference list should be mentioned in the rebuttal letter that accompanies your revised manuscript. If you need to cite a retracted article, indicate the article’s retracted status in the References list and also include a citation and full reference for the retraction notice.

One reference [35] was updated from a pre-print to subsequent publication in PLOS ONE.

Additional Editor Comments (if provided):

Please address comments by reviewer 2 in detail.

See responses below. As noted in my response to the reviewer’s final request, I did include the following text: “Code and data are available on zenodo: <https://doi.org/10.5281/zenodo.5020233>” in the PLOS ONE submission metadata under the assumption that this text will appear as part of the published article. If I need to include this text explicitly in the paper somehow, please let me know.

[Note: HTML markup is below. Please do not edit.]

Reviewers' comments:

Reviewer's Responses to Questions

**Comments to the Author**

1. If the authors have adequately addressed your comments raised in a previous round of review and you feel that this manuscript is now acceptable for publication, you may indicate that here to bypass the “Comments to the Author” section, enter your conflict of interest statement in the “Confidential to Editor” section, and submit your "Accept" recommendation.

Reviewer #1: All comments have been addressed

Reviewer #2: All comments have been addressed

2. Is the manuscript technically sound, and do the data support the conclusions?

The manuscript must describe a technically sound piece of scientific research with data that supports the conclusions. Experiments must have been conducted rigorously, with appropriate controls, replication, and sample sizes. The conclusions must be drawn appropriately based on the data presented.

Reviewer #1: Yes

Reviewer #2: Yes

3. Has the statistical analysis been performed appropriately and rigorously?

Reviewer #1: Yes

Reviewer #2: Yes

4. Have the authors made all data underlying the findings in their manuscript fully available?

The [PLOS Data policy](http://www.plosone.org/static/policies.action#sharing) requires authors to make all data underlying the findings described in their manuscript fully available without restriction, with rare exception (please refer to the Data Availability Statement in the manuscript PDF file). The data should be provided as part of the manuscript or its supporting information, or deposited to a public repository. For example, in addition to summary statistics, the data points behind means, medians and variance measures should be available. If there are restrictions on publicly sharing data—e.g. participant privacy or use of data from a third party—those must be specified.

Reviewer #1: Yes

Reviewer #2: Yes

5. Is the manuscript presented in an intelligible fashion and written in standard English?

PLOS ONE does not copyedit accepted manuscripts, so the language in submitted articles must be clear, correct, and unambiguous. Any typographical or grammatical errors should be corrected at revision, so please note any specific errors here.

Reviewer #1: Yes

Reviewer #2: Yes

6. Review Comments to the Author

Please use the space provided to explain your answers to the questions above. You may also include additional comments for the author, including concerns about dual publication, research ethics, or publication ethics. (Please upload your review as an attachment if it exceeds 20,000 characters)

Reviewer #1: The author addressed all comments from the previous round of reviewing. I recommend this interesting and important study for publication.

Reviewer #2: As a general, rather personal note, I suggest that the author rethink one last time the balance of content between the main manuscript and S1. The text in the manuscript is excessively indirect and narrative, explaining model characteristics and behaviours in a rather vague and wordy style. Almost no reference to the model implementation, experimental setting and output analysis is made before jumping into results. In contrast, the text in S1 explains quite clearly some critical points about the model and its results. Additionally, as it is, S1 is almost a second paper, yet grossly repeating several points already made in the main text. As a reader and someone that also use simulation in this field, I would insist, at least, that the author brings more of S1 content to the main manuscript, at his discretion.

This is, however, aimed at raising the quality of the final text, and not a call-out for a third review round.

While I appreciate and completely understand the reviewer's comments, I deliberately chose, from the outset, to pursue a more narrative approach in the main text to motivate and describe model behaviors and avoid the mathematical and algorithmic details­­­­­—leaving that precision (and duplication of material) to the SI—in order to make the main article more approachable by a wider audience. My previous attempts to consistently and uniformly incorporate model details from the SI into the main text rapidly led to an overly technical Materials and Methods section, which I wanted to avoid. So, however successful I may have been in achieving my narrative goal, at this point I think it best to decline to modify the text as requested.

\# minor suggestions about format

- lines 89, 426, 658: a-historical -> ahistorical

Fixed.

- lines 150, 155, 165, 512: with "net birthrate", "net effective birthrate", or "birth rate", I believe the author means the "population natural growth" or "population natural change", that is, population change that accounts for births and deaths but not migration flows. E.g., see: <https://ec.europa.eu/eurostat/statistics-explained/index.php?title=Glossary:Natural_population_change>

Correct; text improved.

- lines 176-177, Fig 1 caption: "Steppe shown in light blue" is not needed since this is already clear in the legend.

Fixed.

- line 344: each another -> each other

Fixed.

- Fig 3, lines 384-386, and Fig 3 caption: Fig 3 and its description is still quite confusing while also repetitive. The titles added to the figure are not intuitive ("1000 years ending 500BCE"), and the panel reference as left-right and rows as letters only make things more difficult to interxpret. I suggest modifying this figure by adding short concise titles for each row and column, treating it as a 4x2 table, and saving the reader the trouble of deciphering a detailed description in the text. As I see it, these would be: "1500-500BCE", "500BCE-500CE", "500CE-1500CE", "1500BCE-1500CE", for rows; "historical" and "simulated", for columns. If PLOS editorial requires numbering each subplot, I would add letters for each one and not only for rows (e.g., A to H, or A.1 to D.2), but would still have row and columns titles. Last, please make sure that all text is large enough to be legible.

I changed the captions of the different panels in Figure 3, increased the font size and weight for the color bar caption, and added ‘Historical’ and ‘Predicted’ annotations to each. I did not change the manuscript or caption text involving this figure. Figures in SI have been updated as well.

- Fig 4: this figure is really four different plots unless they are reordered so that they share the same x-axis (time). Of course, this would only make sense if the author intends to call the reader's attention to trends in each variable at specific periods. In any case, it would make it much easier to read if legends were included.

I added the date x-axis annotations for the upper panels (removed originally to reduce clutter) and added legends to all panels. Figures in SI have been updated as well.

- Fig 7 and 8 captions: these are unnecessarily long and describe information that can be better shown directly on the figure. There is no need to explain colors if there is a clear legend title and labels. I would suggest the same approach I recommend for Fig 3, i.e., adding clear and concise titles per row and column.

I believe the existing legends and titles, with the accompanying figure captions, are sufficiently clear. I made no changes to these figures or captions.

- Regarding the Zenodo repository: please make sure to add a reference to the paper once published and that the paper refers to the DOI of the latest version or ALL versions. Also, I recommend adding a short description of the repository in Zenodo -- even if only a sentence or two.

I did include the following text: “Code and data are available on zenodo: <https://doi.org/10.5281/zenodo.5020233>” in the PLOS ONE submission metadata under the assumption that this will appear as part of the published article. I will update the PLOS ONE submission metadata with the updated zenodo DOI: <https://doi.org/10.5281/zenodo.5748186>. Of course I will ensure the zendo repository (and the original SocAriv pre-print) point to the published PLOSONE DOI. I updated the zenodo repository to include a better description (based on the paper abstract).

Congratulations and best of luck!

Thank you for all your detailed and thoughtful comments.

7. PLOS authors have the option to publish the peer review history of their article ([what does this mean?](https://journals.plos.org/plosone/s/editorial-and-peer-review-process#loc-peer-review-history)). If published, this will include your full peer review and any attached files.


If you choose “no”, your identity will remain anonymous but your review may still be made public.


**Do you want your identity to be public for this peer review?** For information about this choice, including consent withdrawal, please see our [Privacy Policy](https://www.plos.org/privacy-policy).

Reviewer #1: No

Reviewer #2: **Yes:** Andreas Angourakis
